# Endurance swimming performance and physiology of juvenile Green Sturgeon (*Acipenser medirostris*) at different temperatures

**DOI:** 10.1093/conphys/coaf003

**Published:** 2025-02-08

**Authors:** Kelly D Hannan, Anna E Steel, Mikayla R Debarros, Dennis E Cocherell, Sarah E Baird, Nann A Fangue

**Affiliations:** Department of Wildlife, Fish, and Conservation Biology, University of California, Davis, Davis, CA, 95616, USA; Department of Wildlife, Fish, and Conservation Biology, University of California, Davis, Davis, CA, 95616, USA; Department of Wildlife, Fish, and Conservation Biology, University of California, Davis, Davis, CA, 95616, USA; Department of Wildlife, Fish, and Conservation Biology, University of California, Davis, Davis, CA, 95616, USA; Department of Wildlife, Fish, and Conservation Biology, University of California, Davis, Davis, CA, 95616, USA; Department of Wildlife, Fish, and Conservation Biology, University of California, Davis, Davis, CA, 95616, USA

**Keywords:** stress, cortisol, fish, glucose, lactate

## Abstract

Sturgeon are threatened by anthropogenic changes to river systems, including entrainment or impingement at water diversions (i.e. the unwanted passage of fish through a water intake or physical contact with a barrier screen, likely caused by high intake velocities). Though there are no universally accepted protocols to determine water diversion risk, previous studies on sturgeon suggest that laboratory evaluations of swimming performance are an effective way to describe susceptibility to entrainment or impingement. The swimming performance of juvenile Green Sturgeon (~5 cm fork length), *Acipenser medirostris*, was quantified for fish acclimated to 13 and 18°C for 2 weeks using fixed water velocity endurance tests. Water velocities ranged from 25 to 55 cm s^−1^, and time-to-fatigue was measured at 5 cm s^−1^ increments. Green Sturgeon were quicker to exhaust at the lower acclimation temperature (13°C) compared to fish acclimated to 18°C, for example at 40 cm s^−1^ 13°C acclimated fish impinged ~7.7 times faster than 18°C acclimated fish and ~41.3 times quicker at water velocities of 45 cm s^−1^. Whole-body cortisol grouped by time-to-fatigue (i.e. sustained swimming: time-to-fatigue >200 min, prolonged swimming: time-to-fatigue between 5 and 200 min, rapid swimming: time-to-fatigue <5 min, and non-swimming: control fish) was highest following the swimming experiment for fish utilizing prolonged swimming strategies regardless of temperature exposure. Furthermore, whole body lactate was elevated in fish utilizing prolonged and rapid swimming strategies compared to sustained and control non-swimming fish. Taken together, when swimming to exhaustion, these results suggest that Green Sturgeon were upregulating stress markers and relying on anaerobic metabolism, although both the above trends were driven by 18°C acclimated fish. The time-to-fatigue data suggest that the risk of entrainment was reduced to zero at water speeds ≤ 29.4 cm s^−1^ for 18°C and ≤ 22.6 cm s^−1^ for 13°C acclimated fish.

## Introduction

Human activities have significantly altered freshwater ecosystems, at times impeding migratory corridors of fishes (Baras and Lucas, 2001). Corridor impediments can be formed by physical passage barriers (Mallen-Cooper and Brand, 2007; Pelicice and Agostinho, 2008), or by water extraction or other flow alteration infrastructure (i.e. hydroelectric dams, large pumping stations and smaller agricultural diversions) that can also have detrimental impacts on fishes. Water diversions have been shown to cause injury and mortality (Kimmerer, 2008; Baumgartner *et al.*, 2009; Grimaldo *et al.*, 2009), reduced fitness (Bennett, 2005; Kimmerer, 2008) and habitat degradation (Drinkwater and Frank, 1994; Kingsford, 2000) for many fish species. Mortality can be caused by direct interaction with water diversions (Kimmerer, 2008; Mussen *et al.*, 2014; Poletto *et al.*, 2015), or indirectly as a result of habitat alterations, degradation and fragmentation (Sheer and Steel, 2006; Heublein *et al.*, 2009; Liermann *et al.*, 2012). Furthermore, in many systems heavily altered by water diversions, the vast majority of diversion pipes remain unmodified and lack fish-protection devices (Herren and Kawasaki, 2001; Moyle and Israel, 2005; Mussen *et al.*, 2014).

The ability of fishes to navigate or avoid water diversion structures is related, in part, to their swimming capacity (Hoover *et al.*, 2005; Swanson *et al.*, 2005; Boysen and Hoover, 2009; Verhille *et al.*, 2014). Endurance swimming studies quantify the amount of time fish can maintain swimming in steady water velocities as well as the metabolic fuels being utilized. Endurance tests have previously been used to estimate the risk of entrainment due to dredging (Hoover *et al.*, 2005, 2011; Boysen and Hoover, 2009). Swimming ability among fishes can vary with differences in anatomy (e.g. tail and fin morphology and position; Webb, 1986; Blake, 2004), physiology and behaviour (Kieffer *et al.*, 2009; May and Kieffer, 2017). Fish swimming is often classified as sustained, prolonged or burst, based on time-to-fatigue data at a given water velocity (Webb, 1975; Beamish, 1978; Hammer, 1995). Each of these swimming classifications utilize different metabolic fuels (Webb, 1975; Beamish, 1978; Hammer, 1995). Sustained swim speeds are relatively low and can be maintained for long periods (i.e. >200 min). These speeds do not result in muscular fatigue, are typically fuelled by aerobic metabolism, and are used in foraging and other routine activities (Webb, 1975; Beamish, 1978; Hammer, 1995). Prolonged speeds can be maintained for intermediate durations (i.e. 0.5–200 min), and use both aerobic and anaerobic metabolism (Webb, 1975; Beamish, 1978; Hammer, 1995). Burst speeds are the highest attainable speeds but can only be maintained for short periods (i.e. <0.5 min) due to accumulation of anaerobic metabolites and muscular fatigue (Webb, 1975; Beamish, 1978; Hammer, 1995). Prolonged and burst speeds are used in prey capture, short-term movements in fast current, predator avoidance and, consequently, can be used to characterize escape speeds from water diversions (Webb, 1975; Beamish, 1978; Hammer, 1995). Both prolonged and burst swimming lead to exhaustion and impingement, which can result in the upregulation of metabolic fuels and activation of the stress-axis (Butler *et al.*, 1986, 1989; McKenzie, 2011; Svalheim *et al.*, 2020).

Activation of the stress-axis (the hypothalamo-pituitary-interrenal axis) in fishes can suppress growth rate, reproductive function and the immune response (Maule *et al.*, 1989; Van Weerd and Komen, 1998; Haddy and Pankhurst, 1999). In teleost fishes, catecholamines and corticosteroids are often upregulated during the primary stress response. Cortisol is the major glucocorticoid (a class of corticosteroids) synthesized in teleost fishes (Idler and Trusco, 1972) and has a role in regulating some secondary components of the stress response (Vijayan *et al.*, 1997; Mommsen *et al.*, 1999). In teleost fishes, cortisol has been shown to increase during swimming and the magnitude of the increase is relative to the swimming speed (Zelnik and Goldspink, 1981). Furthermore, increases in the primary stress response triggers the secondary stress response (e.g. increased plasma glucose, haematocrit, lactate, heart rate, metabolic rate and decreased plasma chloride, sodium potassium liver glycogen, muscle protein). The changes resulting from the secondary stress response typically remain for longer periods of time compared to the primary stress response and assist fish in mobilizing fuels to meet increased energy demands (Portz *et al.*, 2006). Cortisol-dependent up-regulation of glucose synthesis promotes the replenishment of hepatic glycogen stores, which decrease in response to short-term stress, and helps to maintain metabolic resources. Increases in plasma lactate levels are also associated with acute stress and are believed to result from increased anaerobic metabolism in fast glycolytic white muscle fibres during ‘burst’ swimming. In the context of managing fish passage impacts, approaches that minimize stresses associated with water diversions should promote the ability for fish to migrate, grow and maintain population stability (Barton, 2002).

Fish passage in proximity to water diversions has been a concern for scientists and managers for decades, and both physical and non-physical guidance systems (e.g. sound barriers, bubble screens, high intensity light, physical screens) that confer fish protection at water diversions have been primarily developed for anadromous salmonids. Salmonids have been the focus of much of the previous research on impingement (i.e. physical contact with a protective screen) and entrainment (i.e. the unwanted passage of fish through a water intake) by water diversions because they are anadromous and the outmigration of small fishes creates many opportunities for repeated interactions with water diversions (Mussen *et al.*, 2013, 2014; Poletto *et al.*, 2015). However, there are other small anadromous fishes that will also encounter water diversions as they move towards the ocean.

One such group of small anadromous fishes are juvenile sturgeon, which are considered to be weaker and slower swimmers relative to other fishes of similar size (Webb, 1986; Peake *et al.*, 1997; Adams *et al.*, 2003; Peake, 2004; Kieffer *et al.*, 2009; Katopodis *et al.*, 2019), preferring to use energy-saving behaviours such as station holding and substrate skimming to maintain position (Kieffer *et al.*, 2009; Deslauriers *et al.*, 2016; May and Kieffer, 2017). Sturgeon are members of the family Acipenseridae, an early lineage of ray-finned fish, while teleosts, accounting for approximately 96% of all extant fish species, represent a more derived group within the infraclass Teleostei. These differences in habitat preference, body shape, swimming capacity and sensory ability (Peake *et al.*, 1997; Moyle, 2002) suggest that species-specific criteria may be needed for the design and operation of water diversions, as salmon criteria may not be conservative enough to confer protection to different species of fish that will interact with water diversions. In the Sacramento River, CA juvenile Green Sturgeon migrate downstream to brackish or ocean habitats within their first year of life. A conceptual model of early behaviour and migration of Green Sturgeon shows that after fish develop into larvae and begin exogenous foraging they initiate a downstream dispersal that lasts about 12 d (Kynard *et al.*, 2005). Post-migrant larvae and juveniles ca. 22–110 dph actively swim up- and downstream to feed before initiating a downstream migration to wintering sites in the fall (Kynard *et al.*, 2005). Additionally, the Sacramento River is a principle artery for water management and has over 3700 water diversions (CalFish, 2023). One strategy to reduce fish entrainment is screening water diversions. However, the current criteria for fish screens within the critical habitat of the Southern distinct population segment of Green Sturgeon are mainly focused on juvenile salmonids. The criteria states that for actively cleaned screens the approach velocity (i.e. the water velocity being pulled through the water diversion screen) cannot exceed 0.4 cm s^−1^ (12.2 cm s^−1^) and for passive screens it cannot exceed 0.2 cm s^−1^ (6.1 cm s^−1^) where juvenile salmonids are present (NMFS, 2011). However, even with these criteria a large majority of diversions remain unscreened. Thus, juvenile Green Sturgeon will risk entrainment many times as they pass water diversion points during their migrations and holding periods (Verhille *et al.*, 2014).

The aim of this study was to evaluate temperature-dependent swimming capacity and associated biochemical responses of juvenile Green Sturgeon. Green sturgeon are composed of at least two genetically distinct populations (Israel *et al.*, 2009). The Northern distinct population segment (nDPS) is classified as a species of concern by the National Oceanic and Atmospheric Administration of the USA. Whereas, the Southern distinct population segment (sDPS) is classified as threatened under the Endangered Species Act. To examine this, we completed experiments at five velocities and at two water temperatures, measuring time-to-fatigue as well as the physiological changes (plasma cortisol, total protein, glucose and lactate) that accompany the stress response upon completion of the endurance test. We predict that fish acclimated to lower temperatures would fatigue faster at lower speeds due to reduced muscle contractility, and the stress response would be augmented by increased velocities and temperatures. Determining endurance metrics and stress responses for juvenile sturgeon will provide additional information to assist with decisions about operational criteria at screens and diversions to aid in protections of juvenile fish.

## Materials and Methods

### Experimental animals

Green Sturgeon were reared at the Centre for Aquatic Biology and Aquaculture, University of California, Davis (UC Davis). Sturgeon were tank spawned and incubated at ~15°C from F2 generation domestic broodstock in March 2022 following the methodology outlined by Van Eenennaam *et al.* (2001, 2012). The initial nDPS parent generation of Green Sturgeon was obtained through a partnership with the Yurok tribe using their gill-net fishery on the lower Kalamath River. At the onset of exogenous feeding (~15 days post hatch; dph), larvae were fed *ad libitum* with Rangen (Duhl, ID, USA) salmonid starter moist feed and gradually brought up to their rearing temperature of 18°C with a change of ~1°C a day. The rearing temperature was chosen based on prior experiments showing that juvenile nDPS Green Sturgeon have increased growth rates when reared at 16–19°C compared to 11–13°C (Poletto *et al.*, 2018) yet is still representative of environmental river temperatures (Supplementary Table S2). Prior to commencing experimentation, all animals were maintained in three replicate tanks (75 L) supplied with flow-through aerated well water (mean ± SD: 18.2 ± 0.5°C, dissolved oxygen 10 ± 0.7 mg L^−1^). Fish used in swimming experiments were 47–53 dph at the time of experiments (mean ± SD: fork length: 5.0 ± 0.5 cm, mass: 1.2 ± 0.3 g). Larval and juvenile Green Sturgeon are present in areas where substantial water volumes are diverted making them susceptible to entrainment (Heublein *et al.*, 2017). Furthermore, many small-scale unscreened diversions are present in the mainstem Sacramento River, and low flow (e.g. during the recent droughts) may reduce the effectiveness of the few fish protection devices and regulations intended to reduce entrainment (Heublein *et al.*, 2017). All handling, rearing and swimming tests were approved by the UC Davis Institutional Animal Care and Use Committee (protocol number 22622).

### Temperature acclimation

Juvenile Green Sturgeon (*n* = 135) were acclimated to two different temperature treatments (13 and 18°C) for 14 d prior to endurance tests. Fish were transferred to 1 of 4, 19 L tanks per temperature treatment and water temperature was changed at a rate of 1°C per day until target temperatures were reached, starting the 14 d acclimation period. In order to match size classes across the different temperature treatments fish exposed to 18°C began acclimation at 33–34 dph whereas fish acclimated to 13°C began acclimation at 38–39 dph. Temperature was manually controlled and measured daily as was dissolved oxygen using a handheld meter (Handy Polaris 2, OxyGuard, Denmark). Heated or chilled unchlorinated well water was pumped into replicate 120 L sumps (*n* = 2 sumps per treatment) with each sump feeding two 19 L tanks. There were no differences between the four tanks within the 13°C or the 18°C acclimation treatment (temperature: (mean ± SD: 13.1 ± 0.3°C and 18.1 ± 0.5°C; dissolved oxygen: 10.7 ± 0.6 mg l^−1^ and 10.0 ± 0.5 mg l^−1^, respectively; Supplementary Table S1)). Green Sturgeon are thought to spawn from April to July, with the peak generally occurring in May to June (Poytress *et al.*, 2015). Currently, managers seek to maintain Sacramento River water temperatures at Bend Bridge (the upper end of Green Sturgeon spawning habitat) below 13.3°C (56°F) for winter-run Chinook from approximately May to July using cold-water releases from Shasta reservoir (US Environmental Protection Agency, 2003). Thus, the lowest temperature treatment (13°C) was chosen to represent this target. However, the average temperature at the Red Bluff Diversion Dam (RBDD) lower on the Sacramento River is slightly higher than this in April 2022 (hourly mean ± SD: 13.9 ± 1.5°C; Supplementary Table S2), and 5 year April average across 2018–2022 (daily mean ± SD: 13.6 ± 1.4°C), the earliest time of year when juvenile Green Sturgeon would likely be above the RBDD. The RBDD was a seasonally operated water diversion dam known to prevent late migrating Green Sturgeon from reaching suitable spawning habitats on the Sacramento River, CA (Heublein *et al.*, 2009), but was decommissioned and replaced with an in-stream diversion in 2013 to allow for adult passage to spawning grounds. The higher temperature (18°C) was chosen as it represents the average maximum daily temperature seen at RBDD from May–September in 2022 (mean ± SD: 17.5 ± 1.2°C) which is higher than the average over the last 5 years (mean ± SD: 15.4 ± 1.4°C; Supplementary Table S2).

### Swimming experiments

Two sizes of experimental swimming tunnels (Loligo, Viborg, Denmark, www.loligosystems.com) were used (5 L and 30 L) for the swimming tests. In order to maximize replicates and minimize fish size differences among and between groups, acrylic inserts were designed to modify these tunnels and create separate identical lanes (2 lanes in each 5 L tunnel and 3 lanes in the 30 L tunnels) so that each swimming section measured 28 cm (length) × 7 cm (height) × 3.6 cm (width). Flows in each lane were calibrated using a handheld flow meter with vane wheel (cm s^−1^ + 0.7% uncertainty) (Flow measuring instrument HFA, Höntzsch, Germany).

**Table 1:** Fixed water velocities expressed as (cm s^−1^) and (BL s^−1^) used for testing the swimming endurance of juvenile Green Sturgeon acclimated to 13°C or 18°C.

Exposure temperature (°C)	Testing velocity		
	cm s^−1^	approx. BL s^−1^	*n*
13	25	5	10
13	30	6	10
13	35	7	10
13	40	8	10
13	45	9	10
18	35	7	10
18	40	8	10
18	45	9	10
18	50	10	10
18	55	11	10

Experiments lasted up to 200 min. Each fish was only tested once at one velocity.

### Training swim

Prior to experimental tests all fish used in swimming trials (*n* = 100) were exposed to the swimming tunnel for a training swim at their acclimation temperature because pilot tests confirmed that participation increased for fish following a training swim (Steel, personal observation; Davison, 1997; Boysen and Hoover, 2009). The length and weight of each fish was measured to the nearest 0.1 cm and 0.1 g, respectively, prior to being transferred into the tunnels. Fish acclimated to 13°C were habituated for 10 min at 0 cm s^−1^, then 10 min at 10 cm s^−1^, then 10 min at 15 cm s^−1^ and 10 min at 20 cm s^−1^. The fish acclimated to 18°C were habituated for 10 min at 0 cm s^−1^, then 10 min at 10 cm s^−1^, then 10 min at 20 cm s^−1^ and then 10 min at 25 cm s^−1^. Once the final speed of habituation was reached (i.e. 20 cm s^−1^ and 25 cm s ^−1^, for 13°C and 18°C acclimated fish, respectively), the training swim lasted an additional 60 min. The speeds of the habituation period differed slightly as pilot testing showed a difference in swimming ability in the two temperature groups. This slow water velocity ramping protocol allowed fish to exhibit positive rheotaxis (head-first orientation into the direction of water flow) prior to increasing water speed to the final fixed water velocity (Boysen and Hoover, 2009). Following the training swim fish were placed back in their acclimation tanks for a 48 h minimum recovery period prior to the endurance tests. During the training swim 26.4% of fish acclimated to 13°C and 14.4% of fish acclimated to 18°C were non-participants.

### Endurance test

Fish were fasted for 24 h prior to each endurance experiment to ensure a post-absorptive state (Niimi and Beamish, 1974). Fish were swum at their acclimation temperature and the initial habituation period was as described above. There were no non-participants in the endurance trials. Fixed water velocities ranged from 25 to 45 cm s^−1^ for 13°C and 35 to 55 cm s^−1^ for 18°C, in 5 cm s^−1^ increments (Table 1). The desired fixed water velocity was produced by adjusting the speed over a 10 s time period and a stopwatch was used to time the experiment. The total length and weight of fish did not differ across temperature treatments (mean ± SD: fork length: 5.0 ± 0.5 cm, mass: 1.2 ± 0.3 g; 47–53 dph, Supplementary Table S3).

During the endurance tests two swimming behaviours were measured (i) endurance or ‘time-to-fatigue’ (i.e. the length of time a fish was able to maintain its position in flowing water) and (ii) the presence or absence of swimming and station-holding behaviours (Hoover *et al.*, 2011). Occasionally during an experiment, a fish was pushed to the back screen. When this happened, the fish was stimulated using a flat and flexible probe to fan or tap the caudal fin. The number and time of stimulations were recorded. If a fish did not leave the back screen after 30 s of stimulations, the test was ended and the time recorded not including the time spent on the back screen (30 s). Stimulations were possible due to the construction of custom lids that had space for probes and a hinged section at the rear of the lid allowing for immediate removal of fish without disturbing other fish. Otherwise, experiments were also ended for fish that swam for 200 min (sustained swimming). Whole fish were cerebrally percussed and immediately flash frozen in liquid nitrogen upon completion of endurance experiments for later evaluation of physiological parameters. Additional fish from each temperature treatment were cerebrally percussed, and flash frozen to serve as control (non-swimming) fish.

### Biochemical analysis

#### Whole-body homogenization

Due to the small size of juvenile Green Sturgeon whole-body measurements of cortisol, glucose, lactate and total protein were made (King and Berlinsky, 2006; Pasparakis *et al.*, 2022). To standardize for the tissue weight of the fish the head of each frozen fish was removed and the remaining tissue was ground to a fine powder, using a mortar and pestle over liquid nitrogen. Prior to homogenization, the ground fish powder was weighed and kept on dry ice. Each sample was then homogenized in 4 ml ice-cold 1× phosphate-buffered saline (PBS buffer: 137 mM sodium chloride, 2.7 mM potassium chloride, 10 mM disodium phosphate and 1.8 mM monopotassium phosphate and pH 7.4) with the addition of protease inhibitors (Roche Molecular Systems, Inc), using a hand-held homogenizer (PRO Scientific, Oxford, CT). Samples were then split into four aliquots for whole-body cortisol, glucose, lactate and protein analyses. The first aliquot of homogenate (1 ml) for cortisol analysis was added to a 9-ml Pyrex glass tube for same day extraction. The remaining homogenate was centrifuged for 30 min at 14 500 g at 4°C. The supernatant was separated and stored at −80°C for later analysis of glucose, lactate and protein (see below).

#### Cortisol analysis

Cortisol extraction followed methods outlined in Hasenbein *et al.* (2013) and Pasparakis *et al.* (2022) with solution volumes optimized for juvenile Green Sturgeon. Briefly, the cortisol homogenate was spiked with 2.5 ml of diethyl ether and vortexed for 1 min prior to centrifuging for 7 min at 5000 g at 4°C. The supernatant was extracted and transferred to a new 9-ml Pyrex glass tube. This process was repeated two additional times per sample and the resulting supernatant was combined (Cachat *et al.*, 2010; Hasenbein *et al.*, 2013; Pasparakis *et al.*, 2022). Samples were placed in the fume hood until complete diethyl ether evaporation. Following evaporation, samples were resuspended in 200 μl 1 × PBS, vortexed and stored at −80°C until later analysis.

Whole-body cortisol levels were measured using an enzyme immunoassay kit, following the manufacturer’s instructions (Salivary Cortisol Immunoassay, Salimetrics, LLC). Samples were run in duplicate, and cortisol concentrations (μg dl^−1^) were calculated using a four-parameter sigmoid standard curve. Cortisol concentrations were corrected for dilution factor and normalized to ground tissue weight (ng cortisol per g fish).

#### Protein analysis

Protein concentrations of each homogenate were measured using the bicinchoninic acid method (23 225 Pierce, Thermo Fisher Scientific, Inc.). Briefly, 200 μl of working reagent was added to standards and samples (in duplicate) after which the plate was incubated for 30 min at 37°C and read at 562 nm. Protein concentration (μg ml^−1^) was calculated using a linear standard curve. Protein concentrations were corrected for dilution. Protein to weight ratios were calculated by dividing total protein (μg ml^−1^) by weight of the ground fish tissue (g).

#### Glucose and lactate analyses

Glucose (glucose assay kit, MAK263 Sigma-Aldrich) and lactate (lactate assay kit II, MAK065 Sigma-Aldrich) analyses were performed following the manufacturer’s instructions. Briefly, 50 μl of the appropriate Reaction Mix was added to standards and samples (in duplicate) the plates were then incubated covered for 30 min at either 37°C (glucose) or room temperature (lactate) and plates were read at 570 and 450 nm, respectively. Concentrations (ng μl^−1^) were calculated using a linear standard curve using the standards provided. Glucose and lactate values were corrected for dilution and normalized to fish ground tissue weight (ng glucose/lactate per g fish).

Standard curves of all assays had *r*^2^ > 0.98 and CV values <10.

### Statistical analyses

All data are reported as mean ± SD unless otherwise specified. Analyses were conducted in R version 4.2.1 (R Core Team, 2022). For each analysis models (additive or multiplicative) and random effects (testing tunnel, swimming lane, fish weight, fork length, time of day, exposure tank or date of experiment) were chosen based on sample-size adjusted Akaike information criterion tests (AICc) using the MuMin package (Bartoń, 2023). The effect of temperature (13 and 18°C) and fixed water velocity (25, 30, 35, 40, 45, 50 and 55 cm s^−1^) on a response variable [time-to-fatigue (min), whole-body cortisol (ng g^−1^), whole-body total protein (μg g^−1^), whole-body lactate (μg g^−1^) and whole-body glucose (μg g^−1^); Supplementary Table S4] were measured using linear mixed-effects models with a Gaussian distribution from the ‘lme4’ package (version 1.1–29; Bates *et al.*, 2015). If initial model diagnostics indicated violations of assumptions (e.g. non-linearity or heteroscedasticity), the response variable was transformed using a logarithmic (log) or square root (sqrt) transformation, as appropriate (Supplementary Table S4). These transformations were selected based on their suitability for stabilizing variance and linearizing relationships with predictor variables.

The time-to-fatigue data were used to separate fish by swimming type (i.e. sustained, prolonged and rapid; see Table 2) for use in analysis. The effect of temperature (13 and 18°C) and swimming type (sustained, prolonged, rapid and control (non-swimming); Table 2) on the response variable [whole-body cortisol (ng g^−1^), whole-body total protein (μg g^−1^), whole-body lactate (μg g^−1^) and whole-body glucose (μg g^−1^); Supplementary Table S4] measured using linear mixed-effects models with a Gaussian distribution (Supplementary Table S4) from the ‘lme4’ package (version 1.1–29; Bates *et al.*, 2015). Similar to the above, if initial model diagnostics indicated violations of assumptions, the response variable was transformed as appropriate (Supplementary Table S4). Cook’s distance was used to identify influential observations in the dataset that might disproportionately affect the model fit.

**Table 2:** Classification of swimming speeds of fishes defined *a priori* based on and modified from Webb (1975); Beamish (1978); Hammer (1995); Adams *et al.* (1999)

Swimming type	Definition	Time-to-fatigue
Control	Non-swimmers	n/a
Rapid	Adapted from the traditionally used ‘burst’ swimming definition. Utilizes mostly anaerobic metabolism; quickly depletes short-term energy stores; used in predator and entrainment avoidance	≤5 min
Prolonged	Utilizes both aerobic and anaerobic metabolism; results in fatigue	>5 to <200 min
Sustained	Utilizes aerobic metabolism; does not result in muscular fatigue; used in migrations, foraging, routine activities	≥200 min

Additionally, the presence or absence of station holding behaviours was examined by temperature (13 and 18°C) and water/swimming speed (body lengths: BL s^−1^) using a logistic regression model with a binomial distribution (Supplementary Table S5) from the ‘stats’ package (version 4.1.2). The effect of temperature (13 and 18°C) and swimming type (sustained, prolonged, rapid and control (non-swimming); Table 2) on the probability of station holding (as presence absence data) was examined using a generalized linear model with a binomial distribution (Supplementary Table S5).

The ‘emmeans’ package (version 1.7.3; Lenth, 2016) was used to summarize all model outputs, including comparisons of swimming treatments to the non-swimming control, grouped within respective temperatures for physiological metrics where appropriate. These comparisons were performed using *post hoc* tests based on estimated marginal means to endure meaningful contrasts within the interaction terms of the model. All plots were produced using ‘ggplot2’ package (version 3.3.5; Wickham, 2016).

## Results

### Endurance tests

**Figure 1:**
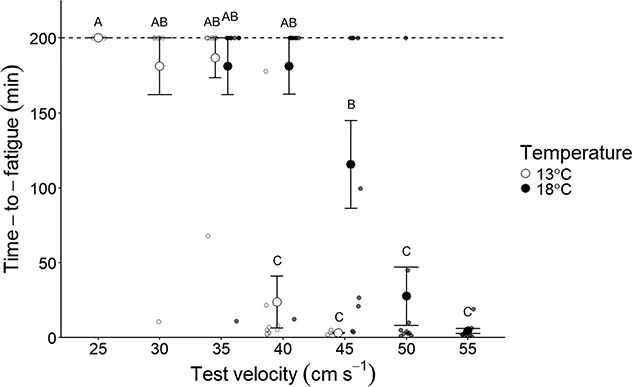
Time-to-fatigue (min) across all fixed water velocities (cm s^−1^) for juvenile *A. medirostris* acclimated to two different temperatures (13°C: white and 18°C: black) for 14 d. The points represent means and error bars represent SE. Raw data are represented by smaller points (*n* = 10 for each treatment). The dotted line represents the end of the endurance test or sustained swimming (200 min). Significant differences between test velocities and temperature treatments are represented by differing letters.

**Figure 2:**
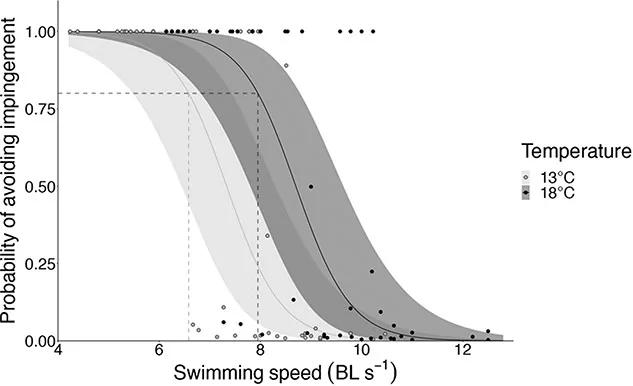
Probability of avoiding impingement during the endurance swimming test (≤200 min) by speed (BL s^−1^) of juvenile *A. medirostris* acclimated to two temperatures (13°C: grey and 18°C: black) for 14 d. A probability of one on the *y*-axis represents completion of the 200-min endurance experiment without impingement. The solid lines represent least-squares means, and the shaded ribbons represent confidence intervals. Points represent raw data. Vertical dashed lines represent 80% probability. Fish exposed to 13 and 18°C are significantly different from each other.

Green Sturgeon acclimated to 13°C had reduced endurance swimming capacity compared to those acclimated to 18°C. This is demonstrated both in the range of swimming velocities tested, chosen based on pilot testing and direct comparisons at 35, 40 and 45 cm s^−1^ (Fig. 1; Supplementary Table S4). Fish acclimated to 13°C were impinged at the back of the swim tunnel 7.7 and 41.3 times more quickly compared to 18°C acclimated fish at 40 and 45 cm s^−1^, respectively (Fig. 1; Supplementary Table S4). Furthermore, when the probability of avoiding impingement (swimming the full 200 min) was compared to water velocity measured in body lengths (BL s^−1^), fish acclimated to 13°C were more likely to impingement at speeds between 6 and 10 BL s^−1^ compared to fish acclimated to 18°C (Fig. 2; Supplementary Table S5). Specifically, 80% of fish were likely to avoid impingement at speeds below 6.58 BL s^−1^ (~33 cm s^−1^) for 13°C acclimated fish, whereas, 80% of 18°C acclimated fish could sustain speeds up to 7.95 BL s^−1^ (~40 cm s^−1^) without impinging (Fig. 2; Supplementary Table S5). The risk of impingement was reduced to zero at water speeds ≤5.88 BL s^−1^ (29.4 cm s^−1^) for 18°C and ≤ 4.52 BL s^−1^ (22.6 cm s^−1^) for 13°C acclimated fish.

### Station holding

**Figure 3:**
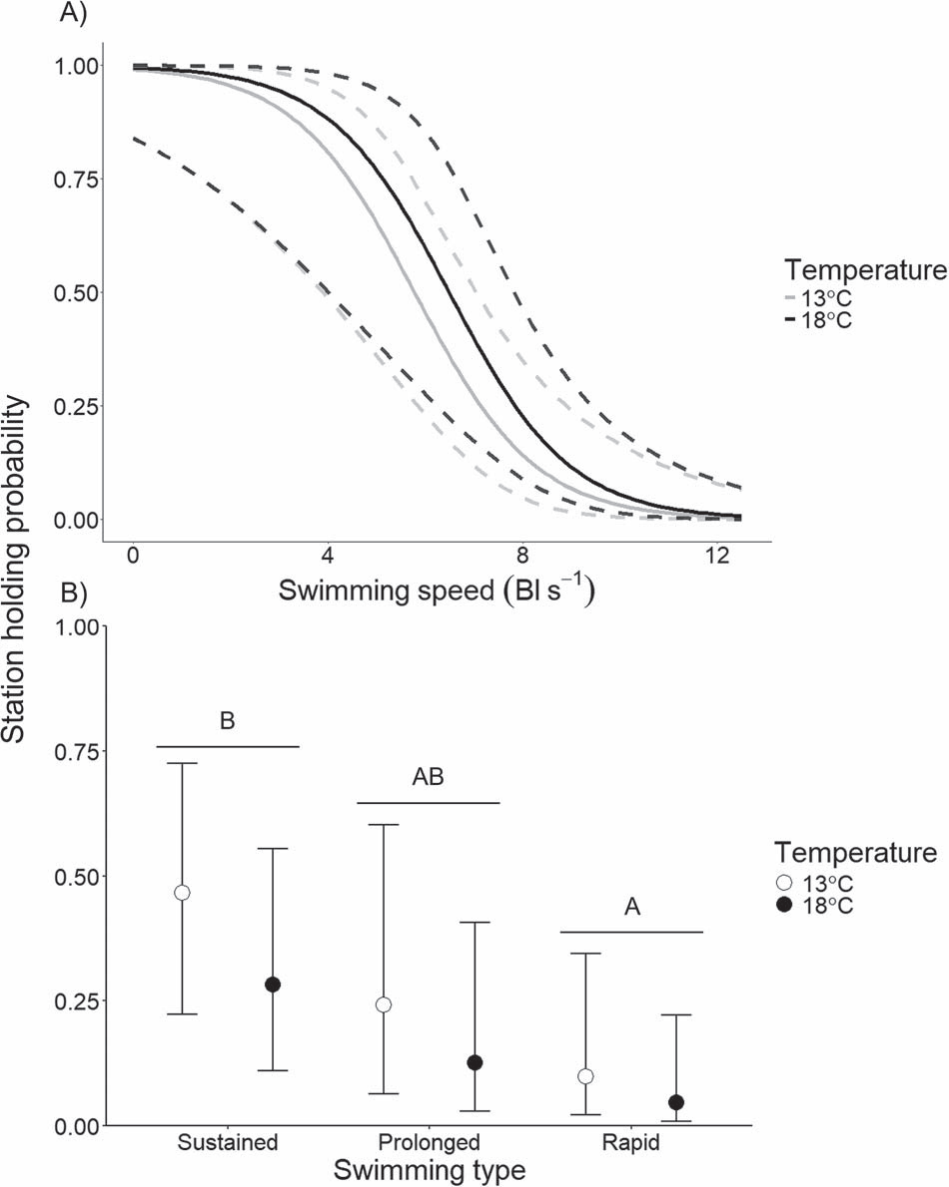
Station holding behaviour derived from presence/absence data represents the likelihood of station holding presence based on (**A**) BL s^−1^ and (**B**) swimming types of juvenile Green Sturgeon acclimated to two temperatures (13°C: grey and 18°C: black) for 14 d. Predicted probabilities are displayed on a continuous scale (0–1). The solid lines represent least-squares means, and the shaded dashed lines represent confidence intervals. Points (3B) represent least-squares means, and error bars represent confidence intervals. Significant differences between swimming types are represented by differing letters.

The probability of station holding decreased with increasing swimming speed (BL s^−1^) and was unaffected by temperature acclimation (Fig. 3A; Supplementary Table S5). When the probability of station holding was compared among fish categorized into each swimming type, it was elevated for sustained swimmers compared to fish exhibiting rapid swimming (Fig. 3B; Supplementary Table S5).

### Cortisol, glucose, lactate and total protein

**Figure 4:**
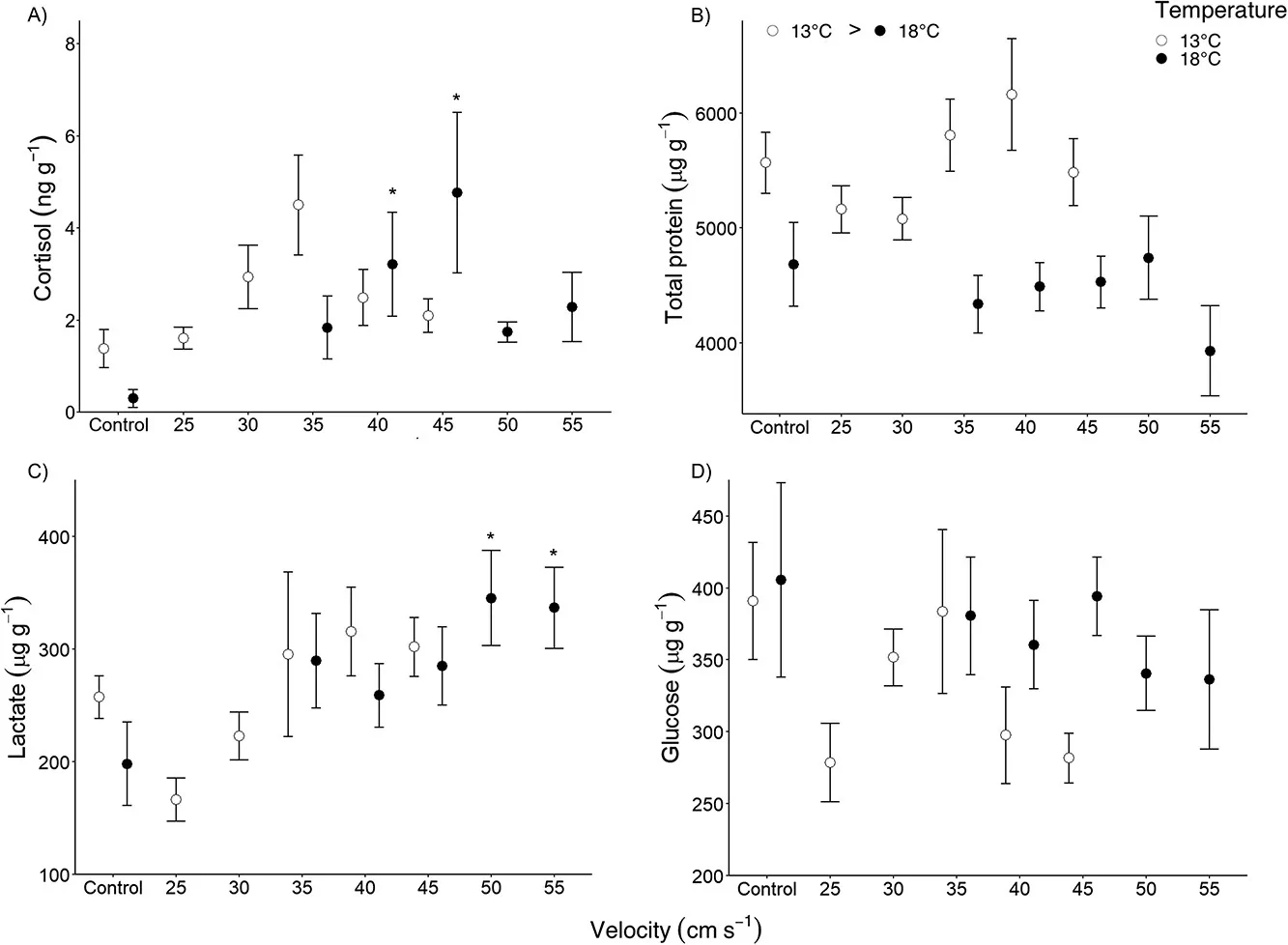
(**A**) Whole-body cortisol, (**B**) whole-body total protein, (**C**) whole-body lactate and (D) whole-body glucose concentrations across water velocity (cm s^−1^) for juvenile *A. medirostris* acclimated to two different temperatures (13°C: white and 18°C: black) for 14 d. Points represent means, and error bars represent standard error. Significant differences between temperatures are represented by a greater-than symbol (>). Differences in velocities compared to relevant temperature controls represented by an asterisk (*).

**Figure 5:**
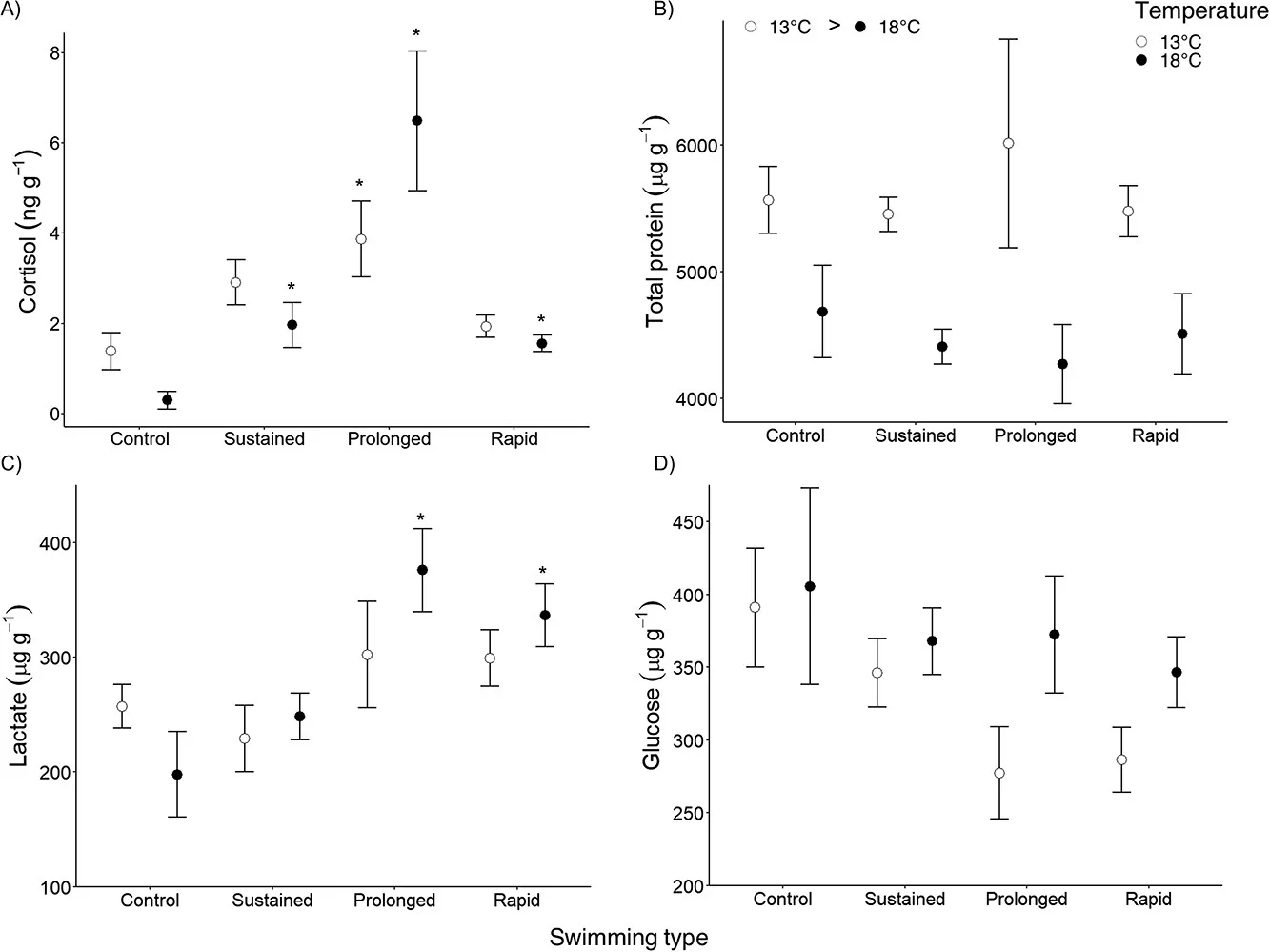
(**A**) Whole-body cortisol, (**B**) whole-body total protein, (**C**) whole-body lactate and (**D**) whole-body glucose concentrations across all swimming types (control: non-swimming, sustained: ≥200 min, prolonged: 5–200 min and rapid: ≤5 min) for juvenile *A. medirostris* acclimated to two different temperatures (13°C: white and 18°C: black) for 14 d. Points represent least-squares means, and error bars represent standard error. Significant differences between temperatures are represented by a greater-than symbol (>) and differences between control and swimming types are represented by asterisks (*).

Whole-body cortisol levels were elevated in fish acclimated to 18°C swimming at mid-velocities (40 and 50 cm s^−1^) compared to non-swimming control fish acclimated to 18°C (Fig. 4A, Supplementary Table S6). However, whole-body cortisol levels in fish acclimated to 13°C were unaffected by velocity and temperature compared to non-swimming control levels (Fig. 4A, Supplementary Table S4/S6). When time-to-fatigue was converted to swimming type, all fish that swam (sustained, prolonged and rapid) and were acclimated to 18°C displayed elevated whole-body cortisol compared to non-swimming control fish, with the peak occurring in prolonged swimming fish (time-to-fatigue: 5–200 min) (Fig. 5A; Supplementary Table S4/S6). In contrast, whole-body cortisol concentrations in fish acclimated to 13°C were elevated only in fish that exhibited prolonged swimming (time-to-fatigue: 5–200 min) compared to non-swimming controls (Fig. 5A; Supplementary Table S4/S6).

Total protein levels were unaffected by both swimming type and water velocity (cm s^−1^) compared to control concentrations but were elevated for fish acclimated to 13°C compared to fish acclimated to 18°C (Figs 4B and 5B; Supplementary Table S4/S6).

Whole-body lactate concentrations were not influenced by temperature acclimation; however, lactate levels varied by water velocity (cm s^−1^) and swimming type (Figs 4C and 5C; Supplementary Table S4). Lactate levels increased with increasing water velocity (cm s^−1^) in fish acclimated to 18°C, with the fastest swimming velocities (50 and 55 cm s^−1^) resulting in elevated lactate compared to non-swimming controls (Fig. 4C, Supplementary Table S6). Although there was an increasing trend with higher water velocity in fish acclimated to 13°C, no significant differences were observed compared to control concentrations for whole-body lactate (Fig. 4C, Supplementary Table S6). The effects of swimming type showed similar trends. Rapid and prolonged swimming fish (i.e. fish that failed before the 200-min experiment was complete) acclimated to 18°C had elevated lactate compared to sustained swimming and control non-swimming fish (Fig. 5C, Supplementary Table S6). However, no differences were observed in fish acclimated to 13°C compared to control non-swimming fish (Fig. 5C; Supplementary Table S4/S6).

Whole-body glucose concentrations in Green Sturgeon were unaffected by water velocity (cm s^−1^), swimming type and temperature acclimation (Figs 4D and 5D; Supplementary Table S4). However, there was a non-significant trend of decreased whole-body glucose for fish exposed to 13°C compared to fish exposed to 18°C when the effects of swimming type and temperature were examined (*P* = 0.067) (Fig. 5D, Supplementary Table S4).

## Discussion

This is the first study to examine endurance swimming in Green Sturgeon. Swimming endurance (time-to-fatigue) increased with increased temperature, suggesting greater risk of impingement/entrainment at colder temperatures. Thus, 5 cm juvenile Green Sturgeon encountering water diversions at 18°C are less likely to become entrained than if they were to encounter the diversion under 13°C conditions, specifically when diversion velocities are within the 40–50 cm s^−1^ range (8–10 BL s^−1^). This suggests the salmon screening criteria, recommending that the approach velocity not exceed 12.2 cm s^−1^ for actively cleaned screens, is protective for Green Sturgeon at 5 cm FL. However, as stated previously there are no regulations on unscreened maximum approach velocities where juvenile salmon are not present, and thus, we recommend that managers implement Green Sturgeon specific maximum approach velocities. Our data, similar to a study on Lake Sturgeon, *A. fulvescens,* shows that the magnitude of the temperature effect is greatest within the prolonged swimming range, and decreases as velocity increases, eventually becoming inconsequential at high speeds (Peake *et al.*, 1997).

### Endurance

Green Sturgeon swam longer at higher temperatures (18°C) when compared to fish acclimated to cooler temperatures (13°C). Furthermore, there were minor differences in acceleration rates between the final acclimation speed and the target fixed swimming velocity between the fish acclimated to 13 and 18°C (0.1–0.5Bl s^−1^ and 0.2–0.6Bl s^−1^, respectively). Although these differences were minor, this could have resulted in an underestimate of swimming in 18°C compared to 13°C acclimated fish as faster acceleration rates are thought to induce fatigue more quickly (May and Kieffer, 2017). If this was the case this would further emphasize the discrepancy in swimming ability between the two temperature acclimations.

The decrease of swimming ability at 13°C is likely associated with decreased muscle contraction (Rome, 1990; Randall and Brauner, 1991). As water velocity and swimming speed increase fish gradually recruit red muscle fibres. Once all of the aerobic fibres have been utilized, any further increase in speed is accomplished through the recruitment of anaerobic fibres. Fish acclimated to cold water will have recruited all their red muscle fibres at a lower water velocity than a fish in warmer water. Thus, the increased speed of recruitment results in the animals reaching fatigue at a lower swimming speed (Randall and Brauner, 1991). This matches the results seen for juvenile Lake Sturgeon where increased temperature exposure enhanced endurance swimming at prolonged swimming speeds (Peake *et al.*, 1997). Additionally, for juvenile Green Sturgeon (~35 cm) Allen *et al.* (2006) found that the maximum sustained swimming speed (*U*_crit_) was 14.9% elevated for fish acclimated to 24°C compared to those acclimated to 19°C. Interestingly, Mayfield and Cech Jr (2004) found the opposite trend for older ~68 cm year 1 juvenile Green Sturgeon, where swimming performance (*U*_crit_) decreased with exposure to 24°C compared to fish acclimated to both 19 and 11°C. This suggests that there may be an ontogenetic or seasonal shift in swimming ability at different temperatures for Green Sturgeon, where early juveniles benefit from increased temperatures. However, this trend has not yet been examined for endurance or burst swimming for this species and further research may provide more information on how the interaction between age and environmental conditions may affect successful passage at diversion sites.

In this study, we were stimulating fish when they became impinged on the back grate of the swimming tunnels, which may have artificially elevated the estimates of passage ability compared to volitional swimming of wild fish. In order to reduce the cost of swimming, sturgeon have adaptations for station holding behaviours. The triangular body form and large pectoral fins allow individuals to press their abdomen and pectoral fins into the substrate to hold position and conserve energy (Deslauriers *et al.*, 2016). Previously, station holding has been shown to extend swimming endurance times in Shortnose Sturgeon (Deslauriers and Kieffer, 2012). Previous studies have hypothesized that sturgeon would increase station holding behaviours as water velocities increased; however, Kieffer *et al.* (2009) found no change in station holding with increased water velocity and further, although not significant, observed increased difficulty station holding at elevated water velocities. This is similar to what we observed in this study. This may be due in part to the smooth walls of the testing chamber within the swim tunnel impeding the fish’s ability to hold position. Indeed, May and Kieffer (2017) observed an increase in station holding behaviour on rough compared to smooth substratum during swimming tests and as speed increased during a *U*_crit_ swimming test. Furthermore, in this study, juvenile sturgeon were not permitted to maintain station holding; however, other studies on Shortnose Sturgeon have found that even with station holding at decreased temperatures Shortnose Sturgeon were unable to compensate for the potential lower muscle output and swimming ability at low temperatures was still decreased compared to higher temperatures (Deslauriers and Kieffer, 2012). Thus, although not measured in this study, we expect that if fish had been permitted to utilize station holding behaviours, it would not benefit endurance swimming at either temperature, and more importantly, fish acclimated to 13°C would still have lower endurance ability compared to fish acclimated to 18°C.

Although this is the first study to examine endurance swimming in Green Sturgeon, there have been at least nine other studies that have examined endurance swimming in other species of sturgeon including Shortnose Sturgeon (Deslauriers and Kieffer, 2011; May and Kieffer, 2017; Kieffer and May, 2020), Pallid Sturgeon (Adams *et al.*, 1999), Lake Sturgeon (Peake *et al.*, 1997), White Sturgeon (Boysen and Hoover, 2009) and Dabry’s Sturgeon (Du *et al.*, 2014) (Table 3). All of these studies have been performed on small juvenile life stages of sturgeon (length < 25 cm) and show that sustained swimming has only been confirmed at 25 cm s^−1^ (~5 BL s^−1^) and below. The results of the endurance tests on juvenile sturgeon reveals that all sturgeons examined are relatively poor swimmers in comparison with both cyprinidae or salmonidae species (Cheong *et al.*, 2006; Kieffer *et al.*, 2009; Deslauriers and Kieffer, 2012; Du *et al.*, 2014).

**Table 3:** Summary of published studies on endurance swimming of juvenile sturgeon species

Study	Species	Endurance velocities tested (cm s^−1^)	Length (cm)	Temperature (°C)	Sustained swimming (cm s^−1^)
Fang *et al.* (2017)	Chinese: *A. sinensis*	~30/36/42/45/52	SL: 9.5–11.4	19	<30
Kieffer and May (2020)	Shortnose: *A. brevirostrum*	25/35/40	TL: 20.4–23.8	15	25
Deslauriers and Kieffer (2011)	Shortnose: *A. brevirostrum*	35/40/45	TL: 19.5	15	<35
Deslauriers and Kieffer (2012)	Shortnose: *A. brevirostrum*	35/40/45	TL: 16	5	<35
Deslauriers and Kieffer (2012)	Shortnose: *A. brevirostrum*	35/40/45	TL: 16	15	<35
Deslauriers and Kieffer (2012)	Shortnose: *A. brevirostrum*	35/40/45	TL: 16	25	<35
May and Kieffer (2017)	Shortnose: *A. brevirostrum*	20/25/30	TL: 18	15	<20
Adams *et al.* (1999)	Pallid: *Scaphirhynchus albus*	5–70	FL: 13–16.8	19	10
Adams *et al.* (1999)	Pallid: *Scaphirhynchus albus*	5–70	FL: 17–20.5	19	25
Peake *et al.* (1997)	Lake: *A. fulvescens*	20/40/45/50	TL: 15	7/14/21	n/a
Peake *et al.* (1997)	Lake: *A. fulvescens*	30–90	TL: 45	7/14/21	n/a
Peake *et al.* (1997)	Lake: *A. fulvescens*	90/120/150/180	TL: 120	7/14/21	n/a
Boysen and Hoover (2009)	White: *A. transmontanus*	30–70	TL: 6.5–8.1	20	<30
Boysen and Hoover (2009)	White: *A. transmontanus*	30–75	TL: 8.2–9.1	20	<30
Boysen and Hoover (2009)	White: *A. transmontanus*	35–80	TL: >9.3	20	<35
Boysen and Hoover (2009)	White: *A. transmontanus*	50–80	TL: >9.3^a^	20	<50
Du *et al.* (2014)	Dabry’s: *A. dabryanus*	20	FL: 5.4–5.9	19	<20
This study	Green: *A. medirostris*	25/30/35/40/45/50/55	FL: 5.0^a^	13	25
This study	Green: *A. medirostris*	25/30/35/40/45/50/55	FL: 5.0^a^	19	<35

TL: total length, SL: standard length, FL: fork length.

a ^a^Fish received training swim

Decreases in swimming performance are thought to arise from increased drag due to the sturgeon spindle-shaped body form and rough body surface (Webb, 1986), in comparison with the smooth surfaces and streamlined fusiform body shape of more derived fishes. Notably, using domesticated sturgeon could affect the observed swimming results as domestication is associated with reduced burst swimming performance for rainbow trout (*Oncorhynchus mykiss*) mainly due to increased body size (Bellinger *et al.*, 2014). So though not explicitly studied there is the potential that domestication could be negatively impacting the observed results, though the fact that these results fall well within the range of other endurance swimming results for similar sized sturgeon is encouraging (Table 3). Furthermore, although it is known that sturgeon have decreased swimming capacity compared to salmonids, the majority of the screening criteria and management plans are created based on salmon data. Reduced swimming capacity of juvenile sturgeon suggests that management plans in regions with sturgeon should be based on sturgeon-specific data to make management decisions that strive for safe passage for all fish species.

### Physiological responses

Resting and stressor-induced concentrations of cortisol in Green Sturgeon are known to be on the low end of the ‘normal’ ranges reported in other fishes (Barton, 2002). Even Green Sturgeon’s closest congener, White Sturgeon (*Acipenser transmontanus*), have at least 2-fold elevated diel peak cortisol response to stress compared to Green Sturgeon (Belanger *et al.*, 2001). In response to stress Lake Sturgeon (*A. fulvescens*) have shown larger catecholamine responses compared to cortisol suggesting that catecholamines may respond more vigorously following stress in these ancient fishes (Hare *et al.*, 2015). However, recent work on Atlantic Sturgeon (*A. oxyrinchus*) has shown the expression of key genes in a tissue-specific manner consistent with the classically understood HPI axis signalling seen in more derived bony vertebrates (Shaughnessy *et al.*, 2023). Taken together, this demonstrates that more research is needed to determine if the stress response in sturgeon is reduced or mediated in a different manner than in teleosts.

When examining the acute stress response following endurance tests at different water velocities and temperatures in this study, whole-body cortisol was higher after endurance swimming, regardless of temperature, when fish exhibited prolonged swimming (finished endurance experiment between 5 and 200 min) compared to control non-swimming fish. Furthermore, the peak cortisol response occurred in the prolonged swimming fish compared to all other swimming types regardless of temperature acclimation (control, rapid and sustained). This reflects the results for 18°C acclimated fish where there were increased cortisol at the mid-water velocities (cm s^−1^) compared to control fish and fish swimming at the slowest and fastest water velocities. These findings are likely due to the timing of cortisol peaks following a stressful event. Lankford *et al.* (2003) found that plasma cortisol of young-of-the-year Green Sturgeon peaked 15 min following a stressful event and remained elevated up to 120 min following the event. Thus, although rapid swimming may have also elicited a cortisol response, fish impinged (and were subsequently sampled) within the first 5 min of the endurance experiments, not leaving enough time to observe an increase in cortisol compared to control fish. Fish that exhibited sustained swimming (i.e. never impinged) did not have altered whole-body cortisol compared to control fish suggesting these speeds were not stressful for these fish. Another factor to consider is the timing of the swimming experiments. Lankford *et al.* (2003) found that the peak cortisol of Green Sturgeon following a stressful event was ca. 3-fold higher when measured at night than during the day. All of the swimming experiments in this study were performed during the day, and the juvenile Green Sturgeon in this study appear to have a similar diel peak cortisol response to young-of-the-year Green Sturgeon measured during the day by Lankford *et al.* (2003). Thus, although Green Sturgeon have lower cortisol responses than many fishes there is still an elevated cortisol response to stress. Furthermore, as the stress responses of Green Sturgeon has been shown to be higher at night when these fish are often active, it would be valuable to examine endurance swimming and recovery from impingement at night.

In this study all cold acclimated fish had elevated total protein compared to warm acclimated fish, regardless of water velocity and swimming type. It is known that enzyme molecules (proteins) turn over rapidly within the body, and thus protein synthesis is a critical element of physiological maintenance. The rate of protein synthesis can vary by acclimation temperature and by fish species. In some instances, protein synthesis increases in warm acclimated fish compared to cold acclimated fish (Johnston and Dunn, 1987), yet in warm acclimated carp (*Cyprinus, carpio*) it decreased over time, and in Rainbow Trout (*Salmo gairdneri*) (Loughna and Goldspink, 1985) it remained unchanged. Acclimation to cooler temperatures approaching lower tolerance limits has been shown to change the fuel-use signature in both Nile Tilapia and Rainbow Trout (Alsop and Wood, 1997; Kieffer *et al.*, 1998; Alsop *et al.*, 1999). Where both species show a decrease in protein and lipid use and an increase in carbohydrate use at colder temperatures (Alsop and Wood, 1997; Kieffer *et al.*, 1998; Alsop *et al.*, 1999). Rates of amino acid incorporation into proteins have been shown to be higher in the liver of cold acclimated goldfish (Das and Prosser, 1967), rainbow trout (Dean and Berlin, 1969) and toadfish (Haschemeyer, 1969). Furthermore, temperature exposure can alter enzyme expression, which may result in increased total protein within the body. For instance, pentose phosphate pathway enzymes increase activity at cold temperatures, corresponding with greater total protein of the liver in Channel Catfish due to either an increase in protein per cell or an increase in total cell number (Seddon and Prosser, 1997). In this study, all cold acclimated fish had elevated total protein compared to warm acclimated fish, regardless of water velocity and swimming type. It is thought that increases in protein synthesis by fish at cold temperatures occurs in an effort to reduce the effects of temperature upon growth and decreases in protein synthesis at high temperatures may be necessary to reduce energy expenditure (Loughna and Goldspink, 1985). Regardless of the mechanism, it seems that juvenile Green Sturgeon are able to modify the amount of whole-body protein based on acclimation temperature, which may be due to altered fuel-use at different temperatures and may increase the functional capacity of the tissues at low temperatures through the increase in enzymatic capacity. However, the levels of total protein were not correlated with swimming endurance time or swimming type.

Plasma/muscle lactate level is an indicator of the anaerobic swimming effort (Black, 1955) and can be useful to indicate a level of fatigue/exhaustion (Farrell *et al.*, 1998). Notably the results of this study are whole-body metrics, which is a holistic overview across the organism. However, this approach cannot differentiate between sources, such as plasma, muscle or liver. There is also a potential for metabolically active tissues to be diluted with tissues with low metabolic activity resulting in lower whole-body results compared to tissue-specific results alone. In Shortnose Sturgeon, muscle lactate typically takes longer to clear from the body following a swimming challenge compared to plasma lactate, (2 and 6 h, respectively), additionally sturgeon peak lactate concentrations in muscle and plasma are typically much lower compared to most other teleost of a similar size (Kieffer *et al.*, 2001). Green sturgeon exposed to 18°C in this study saw increases in whole-body lactate as water velocity increased compared to control values. Sustained swimming utilizes aerobic metabolism and does not result in muscular fatigue. This type of swimming is typically used in migrations, foraging and routine activities. The results of this study show that whole-body lactate concentrations immediately following the sustained swimming experiment are the same as whole-body lactate concentrations of control non-swimming fish regardless of temperature acclimation, which is what we would expect as these fish should have been utilizing aerobic metabolism to maintain swimming at these lower speeds. However, both prolonged and rapid swimming are thought to utilize aerobic and anaerobic metabolism and results in fatigue. The results of this study corroborate this for fish acclimated to 18°C.

However, fish exposed to 13°C showed no differences in lactate across all swimming types. Similar results, where decreased temperatures resulted in much lower lactate mobilization and quicker recovery compared to warmer temperatures, have been seen in Rainbow Trout during repeat *U*_crit_ tests as well as Atlantic Salmon that were angled (Wilkie *et al.*, 1996; Jain and Farrell, 2003). A review on exhaustive exercise in fish found that differences in acclimation temperature mainly affects a fish’s ability to recover after exhaustive swimming rather than the stores of metabolic fuels (Kieffer, 2000). Similarly, a study on the stress responses of larger Green Sturgeon found that in response to air-emersion warm acclimated fish (19°C) had higher spikes in lactate compared to cold acclimated fish (11°C) and the cold acclimated fish took longer to recover in terms of plasma lactate (Lankford *et al.*, 2003). Elevated lactate has also been linked with the inhibition of repeat swimming (Jain and Farrell, 2003). Thus, if cold acclimated fish need to perform repeated swims it may be ecologically beneficial to conserve energy where cold acclimated fish could opt to stop swimming sooner than warm acclimated fish to preserve glycogen reserves (Kieffer, 2000; Jain and Farrell, 2003). Based on this information, one oversimplified explanation for the increased endurance swimming times of warm acclimated fish in this study compared to cold acclimated fish is a greater involvement of anaerobic swimming, given the significantly larger increases in whole-body metabolites observed for warm-acclimated sturgeon. The cold acclimated fish may have been less motivated to swim in order to recover quicker in the event of repeat swimming and preserve fuel reserves.

Glucose is another important metabolic fuel source in most vertebrate animals. Elevated plasma glucose is typically considered part of the secondary stress response, and is often accompanied by decreases in glycogen due to increased maintenance demands. However, there have been mixed findings for the relationship between elevations in plasma glucose and plasma cortisol (Gesto *et al.*, 2014; López-Luna *et al.*, 2016). In this study whole-body glucose concentrations were unaffected by temperature acclimation, water velocity and swimming type. The plasma glucose concentrations of both Shortnose and Atlantic Sturgeon were also unaffected by forced activity (Baker *et al.*, 2005). Similarly, plasma glucose levels of white sturgeon were unaffected by swimming immediately following the swimming challenge (Cocherell *et al.*, 2011). Finally, for larger Green Sturgeon, there were no changes in plasma glucose immediately following air-emersion for 19°C acclimated fish and elevations in plasma glucose were not observed until 20 min post-stressor for 11°C acclimated fish (Lankford *et al.*, 2003). Thus, sturgeon appear robust to changes in glucose caused by exercise and to a lesser extent stress. This could show that sturgeon rely on different metabolic fuels to sustain swimming. However, the whole-body measurements in our study have the potential to mute our ability to detect changes in plasma glucose.

In conclusion, Green Sturgeon displayed differences in endurance swimming ability when acclimated to different water temperatures. However, it is important to note that only two temperature treatments were examined in this study and additional temperatures within and beyond optimal growth would provide further context. Poletto *et al.* (2018) found that Green Sturgeon juveniles have increased growth at higher temperatures (16 and 19°C) compared to cooler temperatures (11 and 13°C) and Mayfield and Cech Jr (2004) found that 15–19°C appeared to be the optimal growth range for larger juvenile fish (144 dph), although higher temperatures were not studied. Finally, Allen *et al.* (2006) found that with abundant food and oxygen the highest temperatures measured (24°C) resulted in the highest growth rates between the ages of 35 and 65 dph. Thus, as the rivers heat up examining swimming at higher temperatures that what is currently occurring would be beneficial for future predictions. The physiological metrics measured in this study suggest that fish raised at an elevated temperature (18°C) were able to maintain swimming at fixed water velocities longer and to utilize more anaerobic fuels than fish at a lower temperature (13°C); however, the fish raised at an elevated temperature potentially required more energy to sustain growth, and if warm-acclimated fish rely heavily on anaerobic metabolism they have the potential to recover more slowly from exercise due to increased accumulation of metabolic end-products (Kieffer, 2000). Fish that were acclimated to 18°C and utilized prolonged swimming (5–200 min), had elevated lactate and cortisol compared to controls suggesting that fish were relying on anaerobic metabolism and experiencing more physiological stress than other swimming types. Fish utilizing rapid swimming strategies were also using anaerobic metabolism, and it is likely they were also experiencing stress; however, the timing of sample collection prevented observed differences in cortisol.

### Managing water diversions for Green Sturgeon

Green Sturgeon juveniles may remain in fresh water for up to 1.5 years before entering seawater (Allen *et al.*, 2009). Because of this long residence time, juveniles likely experience numerous interactions with active water-diversion pipes and fish protective screening. The number of water diversions located throughout the Sacramento–San Joaquin watershed in California’s Central Valley exceeds 3700, and the majority of these are unscreened (CalFish, 2023). Behaviourally, sturgeon are thought to be at a higher risk of entrainment as they prefer to station hold and conserve energy where possible. Physiologically, Lake Sturgeon have been shown to have few superficial neuromasts, the sensory organs in the lateral line that detect changes in water velocity around the fish (Gibbs and Northcutt, 2004), which are frequently more numerous in other taxa (Mogdans, 2022). If this is consistent across sturgeon species, this reduced ability to detect water-diversion inflows compared to other fishes could result in entrainment. Results from the current study suggest that 80% of juvenile sturgeon could successfully pass within close proximity to diversions with intake velocities lower than 34 cm s^−1^ even when river or estuary conditions are at the lowest temperature measured (13°C). However, Mussen *et al.* (2014) found that at slower water velocities (28 cm s^−1^ intake/approach speed and 15 cm s^−1^ river/sweeping speed at 18–19°C) 22.3% of juvenile Green Sturgeon (FL: 35 cm) could become entrained if they pass within 1.5 m of an unscreened diversion pipe. A study by Verhille *et al.* (2014) suggests that night-time flows at water-diversions in the upper and middle reaches of the Sacramento River from May through the summer should be limited to 29 cm s^−1^ when younger, migrating Green Sturgeon are likely to be present. Further, although about 80% of juvenile sturgeon in this study (FL: 5 cm) were able to maintain sustained swimming (≥200 min) at speeds below 34 cm s^−1^, the number of entrainments has been shown to increase with concurrent pipe passages. Encouragingly, Kieffer and May (2020) have seen that juvenile Shortnose Sturgeon (*A. brevirostrum*) are able to perform repeat U_crit_ and endurance swims with 30 and 60 min of recovery periods, respectively, with no difference in performance suggesting a high recovery capacity. Future studies on water diversions would benefit from testing repeat swimming. Currently, to support increased survival management plans should focus on water velocities that allow for 100% passage rates. In our study, 100% of the juvenile Green Sturgeon acclimated to 13°C were able to complete the 200 min endurance test at water velocities of 25 cm s^−1^. However, the model examining the probability of avoiding impingement (swimming the full 200 min) was compared to water velocity measured in body lengths (BL s^−1^), showed that the risk of impingement was reduced to zero at water speeds ≤5.88 BL s^−1^ (29.4 cm s^−1^) for 18°C and ≤ 4.52 BL s^−1^ (22.6 cm s^−1^) for 13°C acclimated fish. This combined with the finding from Mussen *et al.* (2014), which found that larger juvenile Green Sturgeon had >20% chance of entrainment at diversion intake flows of 28 cm s^−1^ suggests that velocities should not exceed 22 cm s^−1^ when designing water diversions that will be unscreened. Screening at water diversions can be used to offer protection for juvenile fishes and prevent entrainment. The current criteria for fish screens within the critical habitat of the sDPS Green Sturgeon state that the approach velocity (i.e. the water velocity being pulled through the water diversion) of active screens (automatically cleaned) cannot exceed 0.4 cm s^−1^ (12.2 cm s^−1^) and passive screens (not automatically cleaned) cannot exceed 0.2 cm s^−1^ (6.1 cm s^−1^) (NMFS, 2011). Although these regulations were created with salmonids in mind, these speeds also should confer protection to the 5 cm sturgeon examined in this study exposed to both 18 and 13°C. However, the vast majority of water diversions are unscreened, and there are currently no criteria on maximum approach velocities for unscreened water diversions, which have the potential to be the most detrimental to fishes especially at the larval and juvenile stages. Based on this research we recommend that for unscreened water diversions the approach velocity not exceed 22 cm s^−1^ (0.7 ft s^−1^) especially when the water temperatures are at or below 13°C. Furthermore, we suggest that management decisions be made using information about fish with the lowest swimming ability (i.e. sturgeon) and that the water temperature be considered when making regulations as this has been shown to affect swimming.

## Supplementary Material

Web_Material_coaf003

## Data Availability

Data used in this study are archived in the Environmental Data Initiative repository (EDI) https://doi.org/10.6073/pasta/e3e172c97f9d3cc32e2680af1faeb2ab

## References

[ref1] Adams SR, Adams GL, Parsons GR (2003) Critical swimming speed and behavior of juvenile shovelnose sturgeon and pallid sturgeon. Trans Am Fish Soc 132: 392–397. 10.1577/1548-8659(2003)132<0392:CSSABO>2.0.CO;2.

[ref2] Adams SR, Hoover JJ, Killgore KJ (1999) Swimming endurance of juvenile pallid sturgeon, *Scaphirhynchus albus*. J Freshwater Ecol 3: 802–807.

[ref3] Allen PJ, Hobbs JA, Cech JJ, Van Eenennaam JP, Doroshov SI (2009) Using trace elements in pectoral fin rays to assess life history movements in sturgeon: estimating age at initial seawater entry in Klamath River green sturgeon. Trans Am Fish Soc 138: 240–250. 10.1577/T08-061.1.

[ref4] Allen PJ, Nicholl M, Cole S, Vlazny A, Cech JJ (2006) Growth of larval to juvenile green sturgeon in elevated temperature regimes. Trans Am Fish Soc 135: 89–96. 10.1577/T05-020.1.

[ref5] Alsop DH, Kieffer JD, Wood CM (1999) The effects of temperature and swimming speed on instantaneous fuel use and nitrogenous waste excretion of the Nile tilapia. Physiol Biochem Zool 72: 474–483. 10.1086/316686. 10438675

[ref6] Alsop DH, Wood CM (1997) The interactive effects of feeding and exercise on oxygen consumption, swimming performance and protein usage in juvenile rainbow trout (*Oncorhynchus mykiss*). J Exp Biol 200: 2337–2346. 10.1242/jeb.200.17.2337. 9320259

[ref107] Baker, DW, Wood, AM, Litvak, MK, Kieffer, JD (2005). Haematology of juvenile Acipenser oxyrinchus and Acipenser brevirostrum at rest and following forced activity. Journal of Fish Biology, 66(1), 208–221. Portico. 10.1111/j.0022-1112.2005.00595.x.

[ref7] Baras E, Lucas MC (2001) Impacts of man’s modifications of river hydrology on the migration of freshwater fishes: a mechanistic perspective. Ecohydrol Hydrobiol 1: 291–304.

[ref8] Barton BA (2002) Stress in fishes: a diversity of responses with particular reference to changes in circulating corticosteroids. Integr Comp Biol 42: 517–525. 10.1093/icb/42.3.517. 21708747

[ref9] Bartoń K (2023) MuMIn: multi-model inference. R Package Version 1475.

[ref10] Bates D, Mächler M, Bolker BM, Walker SC (2015) Fitting linear mixed-effects models using lme4. J Stat Softw 67: 1–48. 10.18637/jss.v067.i01.

[ref11] Baumgartner LJ, Reynoldson NK, Cameron L, Stanger J (2009) Effects of irrigation pumps on riverine fish. Fish Manag Ecol 16: 429–437. 10.1111/j.1365-2400.2009.00693.x.

[ref12] Beamish FWH (1978) Swimming capacity. In WS Hoar, DJ Randall, eds, Fish Physiology. Academic Press, New York, pp. 101–187

[ref13] Belanger JM, Son JH, Laugero KD, Moberg GP, Doroshov SI, Lankford SE, Cech JJ (2001) Effects of short-term management stress and ACTH injections on plasma cortisol levels in cultured white sturgeon, *Acipenser transmontanus*. Aquaculture 203: 165–176. 10.1016/S0044-8486(01)00620-2.

[ref14] Bellinger KL, Thorgaard GH, Carter PA (2014) Domestication is associated with reduced burst swimming performance and increased body size in clonal rainbow trout lines. Aquaculture 420-421: 154–159. 10.1016/j.aquaculture.2013.10.028.

[ref15] Bennett WA (2005) Critical assessment of the Delta smelt population in the San Francisco estuary, California. San Franc Estuary Watershed Sci 3: 1–72. 10.15447/sfews.2005v3iss2art1.

[ref16] Black E (1955) Blood levels of hemoglobin and lactic acid in some freshwater fishes following exercise. J Fish Res Board Can 12: 917–929. 10.1139/f55-048.

[ref17] Blake RW (2004) Fish functional design and swimming performance. J Fish Biol 65: 1193–1222. 10.1111/j.0022-1112.2004.00568.x.

[ref18] Boysen KA, Hoover JJ (2009) Swimming performance of juvenile white sturgeon (*Acipenser transmontanus*): training and the probability of entrainment due to dredging. J Appl Ichthyol 25: 54–59. 10.1111/j.1439-0426.2009.01247.x.

[ref19] Butler PJ, Axelsson M, Ehrenström F, Metcalfe JD, Nilsson S (1989) Circulating catecholamines and swimming performance in the Atlantic cod, *Gadus morhua*. J Exp Biol 141: 377–387. 10.1242/jeb.141.1.377.

[ref20] Butler PJ, Metcalfe JD, Ginley SA (1986) Plasma catecholamines in the lesser spotted dogfish and rainbow trout rest and during different levels of exercise. J Exp Biol 123: 409–421. 10.1242/jeb.123.1.409. 3746197

[ref21] Cachat J, Stewart A, Grossman L, Gaikwad S, Kadri F, Chung KM, Wu N, Wong K, Roy S, Suciu C et al. (2010) Measuring behavioral and endocrine responses to novelty stress in adult zebrafish. Nat Protoc 5: 1786–1799. 10.1038/nprot.2010.140. 21030954

[ref22] CalFish (2023) A California cooperative anadromous fish and habitat data program. https://www.calfish.org/ProgramsData/HabitatandBarriers/CaliforniaFishPassageAssessmentDatabase.aspx (February 9, 2024, date accessed).

[ref23] Cheong , Kavvas ML, Anderson EK (2006) Evaluation of adult white sturgeon swimming capabilities and applications to fishway design. Environ Biol Fishes 77: 197–208. 10.1007/s10641-006-9071-y.

[ref24] Cocherell DE, Kawabata A, Kratville DW, Cocherell SA, Kaufman RC, Anderson EK, Chen ZQ, Bandeh H, Rotondo MM, Padilla R et al. (2011) Passage performance and physiological stress response of adult white sturgeon ascending a laboratory fishway. J Appl Ichthyol 27: 327–334. 10.1111/j.1439-0426.2010.01650.x.

[ref25] Das AB, Prosser CL (1967) Biochemical changes in tissues of goldfish acclimated to high and low temperatures-I. Protein synthesis. Comp Biochem Physiol 21: 449–467. 10.1016/0010-406X(67)90445-8. 6051649

[ref26] Davison W (1997) The effects of exercise training on teleost fish, a review of recent literature. Comp Biochem Physiol 117: 67–75. 10.1016/S0300-9629(96)00284-8.

[ref27] Dean JM, Berlin JD (1969) Alterations in hepatocyte function of thermally acclimated rainbow trout (*Salmo gairdneri*). Comp Biochem Physiol 29: 307–312. 10.1016/0010-406X(69)91750-2. 5795821

[ref28] Deslauriers D, Johnston R, Chipps SR (2016) Effect of morphological fin-curl on the swimming performance and station-holding ability of juvenile shovelnose sturgeon. J Fish Wildl Manag 7: 198–204. 10.3996/092015-JFWM-087.

[ref29] Deslauriers D, Kieffer JD (2011) The influence of flume length and group size on swimming performance in Shortnose sturgeon *Acipenser brevirostrum*. J Fish Biol 79: 1146–1155. 10.1111/j.1095-8649.2011.03094.x. 22026598

[ref30] Deslauriers D, Kieffer JD (2012) The effects of temperature on swimming performance of juvenile Shortnose sturgeon (*Acipenser brevirostrum*). J Appl Ichthyol 28: 176–181. 10.1111/j.1439-0426.2012.01932.x.

[ref31] Drinkwater KF, Frank KT (1994) Effects of river regulation and diversion on marine fish and invertebrates. Aquat Conserv Mar Freshw Ecosyst 4: 135–151. 10.1002/aqc.3270040205.

[ref32] Du H, Wei QW, Xie X, Shi LL, Wu JM, Qiao XM, Liu ZG (2014) Improving swimming capacity of juvenile Dabry’s sturgeon, (*Acipenser dabryanus* Duméril, 1869) in current-enriched culture tanks. J Appl Ichthyol 30: 1445–1450. 10.1111/jai.12591.

[ref106] Fang M, Cai, L, Gao, Y, He, D, Johnson, D, Huang, Y (2017). Swimming and Recovery Abilities of Juvenile Chinese Sturgeon Acipenser sinensis. Transactions of the American Fisheries Society, 146(6), 1186–1192. Portico. 10.1080/00028487.2017.1362469

[ref33] Farrell AP, Gamperl AK, Birtwell IK (1998) Prolonged swimming, recovery and repeat swimming performance of mature sockeye Salmon *Oncorhynchus nerka* exposed to moderate hypoxia and pentachlorophenol. J Exp Biol 201: 2183–2193. 10.1242/jeb.201.14.2183. 9639592

[ref34] Gesto M, Otero-Rodiño C, López-Patiño MA, Míguez JM, Soengas JL, Conde-Sieira M (2014) Is plasma cortisol response to stress in rainbow trout regulated by catecholamine-induced hyperglycemia? Gen Comp Endocrinol 205: 207–217. 10.1016/j.ygcen.2014.04.002. 24735744

[ref35] Gibbs MA, Northcutt RG (2004) Development of the lateral line system in the shovelnose sturgeon. Brain Behav Evol 64: 70–84. 10.1159/000079117. 15205543

[ref36] Grimaldo LF, Sommer T, Van Ark N, Jones G, Holland E, Moyle PB, Herbold B, Smith P (2009) Factors affecting fish entrainment into massive water diversions in a tidal freshwater estuary: can fish losses be managed? N Am J Fish Manag 29: 1253–1270. 10.1577/M08-062.1.

[ref37] Haddy JA, Pankhurst NW (1999) Stress-induced changes in concentrations of plasma sex steroids in Black bream. J Fish Biol 55: 1304–1316.

[ref38] Hammer C (1995) Fatigue and exercise tests with fish. Comp Biochem Physiol 112: 1–20.

[ref39] Hare AJ, Waheed A, Hare JF, Anderson WG (2015) Cortisol and catecholamine responses to social context and a chemical alarm signal in juvenile lake sturgeon, *Acipenser fulvescens*. Can J Zool 93: 605–613. 10.1139/cjz-2015-0045.

[ref40] Haschemeyer AE (1969) Rates of polypeptide chain assembly in liver in vivo: relation to the mechanism of temperature acclimation in *Opsanus tau*. Proc Natl Acad Sci U S A 62: 128–135. 10.1073/pnas.62.1.128. PMC285964 5253648

[ref41] Hasenbein M, Komoroske LM, Connon RE, Geist J, Fangue NA (2013) Turbidity and salinity affect feeding performance and physiological stress in the endangered Delta smelt. Integr Comp Biol 53: 620–634. 10.1093/icb/ict082. 23922273

[ref42] Herren JR, Kawasaki SS (2001) Inventory of water diversions in four geographic areas in California’s Central Valley. Fish Bulletin 179: 343–355.

[ref43] Heublein J, Bellmer R, Chase RD, Doukakis P, Gingras M, Hampton D, Israel JA, Jackson ZJ, Johnson RC, Langness OP et al. (2017) Improved fisheries management through life stage monitoring: the case for the southern distinct population segment of north American green sturgeon and the Sacramento-San Joaquin River white sturgeon. *NOAA Technical Memorandum NMFS* 35.

[ref44] Heublein JC, Kelly JT, Crocker CE, Klimley AP, Lindley ST (2009) Migration of green sturgeon, *Acipenser medirostris*, in the Sacramento River. Environ Biol Fishes 84: 245–258. 10.1007/s10641-008-9432-9.

[ref45] Hoover JJ, Boysen KA, Beard JA, Smith H (2011) Assessing the risk of entrainment by cutterhead dredges to juvenile Lake sturgeon (*Acipenser fulvescens*) and juvenile pallid sturgeon (*Scaphirhynchus albus*). J Appl Ichthyol 27: 369–375. 10.1111/j.1439-0426.2011.01746.x.

[ref46] Hoover JJ, Killgore KJ, Clarke DG, Smith H, Turnage A, Beard J (2005) Paddlefish and Sturgeon Entrainment by Dredges: Swimming Performance as an Indicator of Risk. U.S. Army Engineer Research and Development Center, Vicksburg, MS

[ref47] Idler D, Trusco B (1972) Corticosteroids in fish. In DR Idler, ed, Steroids in Nonmammalian Vertebrates. Academic Press, New York, pp. 126–252

[ref48] Israel JA, Bando KJ, Anderson EC, May B (2009) Polyploid microsatellite data reveal stock complexity among estuarine north American green sturgeon (*Acipenser medirostris*). Can J Fish Aquat Sci 66: 1491–1504. 10.1139/F09-091.

[ref49] Jain KE, Farrell AP (2003) Influence of seasonal temperature on the repeat swimming performance of rainbow trout *Oncorhynchus mykiss*. J Exp Biol 206: 3569–3579. 10.1242/jeb.00588. 12966048

[ref50] Johnston IA, Dunn J (1987) Temperature acclimation and metabolism in ectotherms with particular reference to teleost fish. Symp Soc Exp Biol 41: 67–93. 3332497

[ref51] Katopodis C, Cai L, Johnson D (2019) Sturgeon survival: the role of swimming performance and fish passage research. Fish Res 212: 162–171. 10.1016/j.fishres.2018.12.027.

[ref52] Kieffer JD (2000) Limits to exhaustive exercise in fish. Comp Biochem Physiol 126: 161–179. 10.1016/S1095-6433(00)00202-6. 10938136

[ref53] Kieffer JD, Alsop D, Wood CM (1998) A Respirometric analysis of fuel use during aerobic swimming at different temperatures in rainbow trout (*Oncorhynchus Mykiss*). J Exp Biol 201: 3123–3133. 10.1242/jeb.201.22.3123. 9787132

[ref54] Kieffer JD, Arsenault LM, Litvak MK (2009) Behaviour and performance of juvenile Shortnose sturgeon *Acipenser brevirostrum* at different water velocities. J Fish Biol 74: 674–682. 10.1111/j.1095-8649.2008.02139.x. 20735587

[ref55] Kieffer JD, May LE (2020) Repeat *U*_Crit_ and endurance swimming in juvenile Shortnose sturgeon (*Acipenser brevirostrum*). J Fish Biol 96: 1379–1387. 10.1111/jfb.14306. 32128813

[ref56] Kieffer JD, Wakefield AM, Litvak MK (2001) Juvenile sturgeon exhibit reduced physiological responses to exercise. J Exp Biol 204: 4281–4289. 10.1242/jeb.204.24.4281. 11815652

[ref57] Kimmerer W (2008) Losses of Sacramento River Chinook Salmon and Delta smelt to entrainment in water diversions in the Sacramento-San Joaquin. San Franc Estuary Watershed Sci 6: 1–27. 10.15447/sfews.2008v6iss2art2.

[ref58] King WV, Berlinsky DL (2006) Whole-body corticosteroid and plasma cortisol concentrations in larval and juvenile Atlantic cod *Gadus morhua* L. following acute stress. Aquacult Res 37: 1282–1289. 10.1111/j.1365-2109.2006.01558.x.

[ref59] Kingsford RT (2000) Ecological impacts of dams, water diversions and river management on floodplain wetlands in Australia. Austral Ecol 25: 109–127. 10.1046/j.1442-9993.2000.01036.x.

[ref60] Kynard B, Parker E, Parker T (2005) Behavior of early life intervals of Klamath River green sturgeon, Acipenser medirostris, with a note on body color. Environ Biol Fishes 72: 85–97. 10.1007/s10641-004-6584-0.

[ref61] Lankford SE, Adams TE, Cech JJ (2003) Time of day and water temperature modify the physiological stress response in green sturgeon, *Acipenser medirostris*. Comp Biochem Physiol 135: 291–302. 10.1016/S1095-6433(03)00075-8. 12781829

[ref62] Lenth R (2016) Least-squares means: the R package lsmeans. J Stat Softw 69: 1–33. 10.18637/jss.v069.i01.

[ref63] Liermann CR, Nilsson C, Robertson J, Ng RY (2012) Implications of dam obstruction for global freshwater fish diversity. Bioscience 62: 539–548. 10.1525/bio.2012.62.6.5.

[ref64] López-Luna J, Bermejo-Poza R, Torrent Bravo F, Villarroel M (2016) Effect of degree-days of fasting stress on rainbow trout, Oncorhynchus mykiss. Aquaculture 462: 109–114. 10.1016/j.aquaculture.2016.05.017.

[ref65] Loughna PT, Goldspink G (1985) Muscle protein synthesis rates during temperature acclimation in a eurythermal (*Cyprinus carpio*) and a stenothermal (*Salmo gairdneri*) species of teleost. J Exp Biol 118: 267–276. 10.1242/jeb.118.1.267.

[ref66] Mallen-Cooper M, Brand DA (2007) Non-salmonids in a salmonid fishway: what do 50 years of data tell us about past and future fish passage? Fish Manag Ecol 14: 319–332. 10.1111/j.1365-2400.2007.00557.x.

[ref67] Maule A, Tripp R, Kaattari S, Schreck C (1989) Stress alters immune function and disease resistance in Chinook Salmon (*Oncorhynchus tshawytscha*). J Endocrinol 120: 135–142. 10.1677/joe.0.1200135. 2918264

[ref68] May LE, Kieffer JD (2017) The effect of substratum type on aspects of swimming performance and behaviour in Shortnose sturgeon *Acipenser brevirostrum*. J Fish Biol 90: 185–200. 10.1111/jfb.13159. 27723098

[ref69] Mayfield RB, Cech JJ Jr (2004) Temperature effects on green sturgeon bioenergetics. Trans Am Fish Soc 133: 961–970. 10.1577/T02-144.1.

[ref70] McKenzie DJ (2011) Swimming and Other Activities | Energetics of Fish Swimming. In AP Farrell, ed, Encyclopedia of Fish Physiology. Elsevier, pp. 1636–1644. 10.1016/b978-0-12-374553-8.00151-9

[ref71] Mogdans J (2022) The processing of hydrodynamic dtimuli with the fish lateral line system. In Oxford Research Encyclopedia of Neuroscience. Oxford University Press. 10.1093/acrefore/9780190264086.013.318

[ref72] Mommsen TP, Vijayan MM, Moon TW (1999) Cortisol in teleosts: dynamics, mechanisms of action, and metabolic regulation. Rev Fish Biol Fish 9: 211–268. 10.1023/A:1008924418720.

[ref73] Moyle PB (2002) Inland Fishes of California. University of California Press, University of California Press, Berkeley, CA

[ref74] Moyle PB, Israel JA (2005) Untested assumptions: effectiveness of screening diversions for conservation of fish populations. Fisheries 30: 20–28. 10.1577/1548-8446(2005)30[20:UA]2.0.CO;2.

[ref75] Mussen TD, Cocherell D, Hockett Z, Ercan A, Bandeh H, Kavvas ML, Chech JJ Jr, Fangue NA (2013) Assessing juvenile Chinook Salmon behaviour and entrainment risk near unscreened water diversions: large flume simulations. Trans Am Fish Soc 142: 130–142. 10.1080/00028487.2012.720633.

[ref76] Mussen TD, Cocherell D, Poletto JB, Reardon JS, Hockett Z, Ercan A, Bandeh H, Levent Kavvas M, Cech JJ, Fangue NA (2014) Unscreened water-diversion pipes pose an entrainment risk to the threatened green sturgeon, *Acipenser medirostris*. PloS One 9: 1–9. 10.1371/journal.pone.0086321. PMC3893286 24454967

[ref77] Niimi AJ, Beamish FWH (1974) Bioenergetics and growth of largemouth bass (*Micropterus salmoides*) in relation to body weight and temperature. Can J Zool 52: 447–456. 10.1139/z74-056. 4832961

[ref78] NMFS (2011) Anadromous Salmon Passage Facility Design. National Marine Fisheries Services, Northwest Region

[ref79] Pasparakis C, Wampler AN, Lohroff T, DeCastro F, Cocherell DE, Carson EW, Hung T-C, Connon RE, Fangue NA, Todgham AE (2022) Characterizing the stress response in juvenile Delta smelt exposed to multiple stressors. Comp Biochem Physiol A Mol Integr Physiol 274: 111303. 10.1016/j.cbpa.2022.111303. 36049729

[ref80] Peake S, Beamish FWH, McKinley RSS, Scruton DAA, Katopodis C (1997) Relating swimming performance of Lake sturgeon, *Acipenser fulvescens*, to fishway design. Can J Fish Aquat Sci 54: 1361–1366. 10.1139/f97-039.

[ref81] Peake SJ (2004) Swimming and respiration. In TO Lebreton, FW Beamish, RS McKinley, eds, Sturgeons and Paddlefish of North America. Kluwer Academic Publishers, Dordrecht, pp. 147–166

[ref82] Pelicice FM, Agostinho AA (2008) Fish-passage facilities as ecological traps in large neotropical rivers. Conserv Biol 22: 180–188. 10.1111/j.1523-1739.2007.00849.x. 18254863

[ref83] Poletto JB, Cocherell DE, Mussen TD, Ercan A, Bandeh H, Levent Kavvas M, Cech JJ, Fangue NA (2015) Fish-protection devices at unscreened water diversions can reduce entrainment: evidence from behavioural laboratory investigations. Conserv Physiol 3: 1–12. 10.1093/conphys/cov040. PMC4778469 27293725

[ref84] Poletto JB, Martin B, Danner E, Baird SE, Cocherell DE, Hamda N, Cech JJ Jr, Fangue NA (2018) Assessment of multiple stressors on the growth of larval green sturgeon *Acipenser medirostris*: implications for recruitment of early life-history stages. J Fish Biol 93: 952–960. 10.1111/jfb.13805. 30246375

[ref85] Portz DE, Woodley CM, Cech JJ (2006) Stress-associated impacts of short-term holding on fishes. Rev Fish Biol Fish 16: 125–170. 10.1007/s11160-006-9012-z.

[ref86] Poytress WR, Gruber JJ, Van Eenennaam JP, Gard M (2015) Spatial and temporal distribution of spawning events and habitat characteristics of Sacramento river green sturgeon. Trans Am Fish Soc 144: 1129–1142. 10.1080/00028487.2015.1069213.

[ref87] R Core Team (2022) R: a language and environment for statistical computing.

[ref88] Randall D, Brauner C (1991) Effects of environmental factors on exercise in fish. J Exp Biol 160: 113–126. 10.1242/jeb.160.1.113.

[ref89] Rome LC (1990) Influence of temperature on muscle recruitment and muscle function in vivo. Am J Physiol 259: R210–R222. 10.1152/ajpregu.1990.259.2.R210. 2201215

[ref90] Seddon WL, Prosser CL (1997) Seasonal variations in the temperature acclimation response of the channel catfish, *Ictalurus punctatus*. Physiol Zool 70: 33–44. 10.1086/639537. 9231374

[ref91] Shaughnessy CA, Myhre VD, Hall DJ, McCormick SD, Dores RM (2023) Hypothalamus-pituitary-interrenal (HPI) axis signaling in Atlantic sturgeon (Acipenser oxyrinchus) and sterlet (Acipenser ruthenus). Gen Comp Endocrinol 339: 114290. 10.1016/j.ygcen.2023.114290. 37088167

[ref92] Sheer MB, Steel EA (2006) Lost watersheds: barriers, aquatic habitat connectivity, and salmon persistence in the Willamette and lower Columbia River basins. Trans Am Fish Soc 135: 1654–1669. 10.1577/T05-221.1.

[ref93] Svalheim RA, Aas-Hansen Ø, Heia K, Karlsson-Drangsholt A, Olsen SH, Johnsen HK (2020) Simulated trawling: exhaustive swimming followed by extreme crowding as contributing reasons to variable fillet quality in trawl-caught Atlantic cod (*Gadus morhua*). PloS One 15: 1–15. 10.1371/journal.pone.0234059. PMC7302710 32555614

[ref94] Swanson C, Young PS, Cech JJ (2005) Close encounters with a fish screen: integrating physiological and behavioral results to protect endangered species in exploited ecosystems. Trans Am Fish Soc 134: 1111–1123. 10.1577/T04-121.1.

[ref95] U.S. Environmental Protection Agency (2003) EPA Region 10 Guidance for Pacifc Northwest State and Tribal Temperature Water Quality Standards. Region 10 Office of Water, Seattle, WA.

[ref96] Van Eenennaam JP, Linares-Casenave J, Doroshov SI (2012) Tank spawning of first generation domestic green sturgeon. J Appl Ichthyol 28: 505–511. 10.1111/j.1439-0426.2012.02012.x.

[ref97] Van Eenennaam JP, Webb MAH, Deng X, Doroshov SI, Mayfield RB, Cech JJ Jr, Hillemeier DC, Willson TE (2001) Artificial spawning and larval rearing of Klamath River green sturgeon. Trans Am Fish Soc 130: 159–165. 10.1577/1548-8659(2001)130<0159:ASALRO>2.0.CO;2.

[ref98] Van Weerd JH, Komen J (1998) The effects of chronic stress on growth in fish: a critical appraisal. Comp Biochem Physiol 120: 107–112. 10.1016/S1095-6433(98)10017-X.

[ref99] Verhille CE, Poletto JB, Cocherell DE, DeCourten B, Baird S, Cech JJ, Fangue NA (2014) Larval green and white sturgeon swimming performance in relation to water-diversion flows. Conserv Physiol 2: 1–14. 10.1093/conphys/cou031. PMC4806727 27293652

[ref100] Vijayan MM, Pereira C, Grau EG, Iwama GK (1997) Metabolic responses associated with confinement stress in tilapia: the role of cortisol. Comp Biochem Physiol 116: 89–95. 10.1016/S0742-8413(96)00124-7.

[ref101] Webb PW (1975) Hydrodynamic and energetics of fish propulsion. Bulletin of the Fisheries Research Board of Canada, pp. 1–158.

[ref102] Webb PW (1986) Kinematics of Lake sturgeon, *Acipenser fulvescens*, at cruising speeds. Can J Zool 64: 2137–2141. 10.1139/z86-328.

[ref103] Wickham H (2016) ggplot2: Elegant graphics for data analysis. Springer, New York

[ref104] Wilkie MP, Davidson K, Brobbel MA, Kieffer JD, Booth RK, Bielak AT, Tufts BL (1996) Physiology and survival of wild Atlantic Salmon following angling in warm summer waters. Trans Am Fish Soc 125: 572–580. 10.1577/1548-8659(1996)125<0572:PASOWA>2.3.CO;2.

[ref105] Zelnik PR, Goldspink G (1981) The effect of exercise on plasma cortisol and blood sugar levels in the rainbow trout, *Salmo gairdnerii* Richardson. J Fish Biol 19: 37–43. 10.1111/j.1095-8649.1981.tb05809.x.

