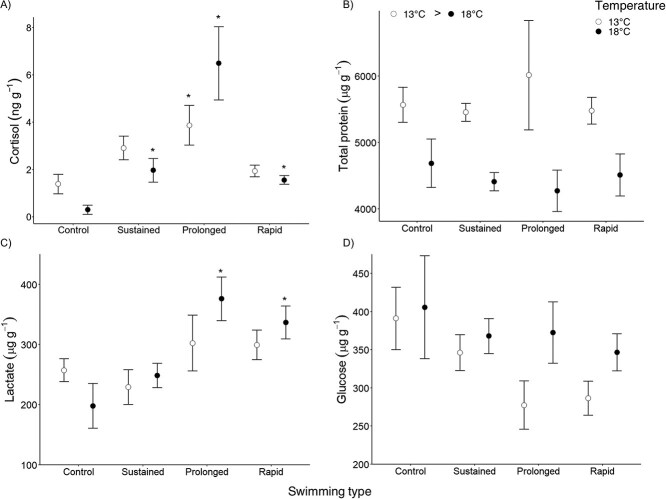# Correction to: Endurance swimming performance and physiology of juvenile Green Sturgeon (*Acipenser medirostris*) at different temperatures

**DOI:** 10.1093/conphys/coag010

**Published:** 2026-02-05

This is a correction to: Kelly D Hannan, Anna E Steel, Mikayla R Debarros, Dennis E Cocherell, Sarah E Baird, Nann A Fangue, Endurance swimming performance and physiology of juvenile Green Sturgeon (*Acipenser medirostris*) at different temperatures, *Conservation Physiology*, Volume 13, Issue 1, 2025, coaf003, 10.1093/conphys/coaf003

The originally published version of this article included incorrect versions of Figure 4A and Figure 5A, in which the cortisol data was presented at a fivefold higher level than intended.

In addition, Editor Caleb Hasler’s name was inadvertently omitted from the HTML version of the article.

These errors have now been corrected, and the published article has been updated accordingly.

**Figure 4:**
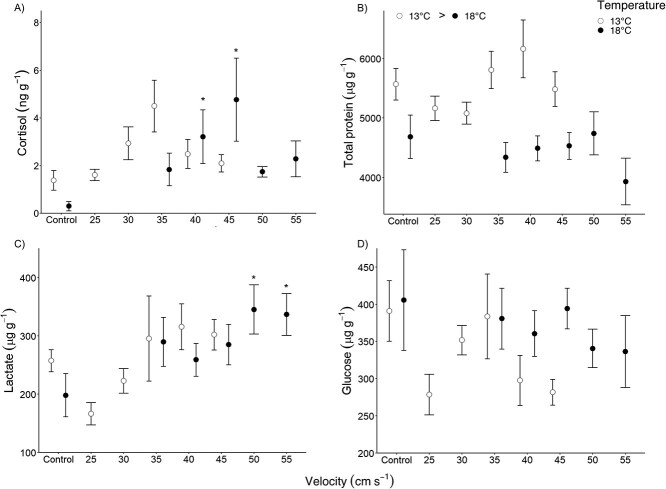


**Figure 5:**