# Supplementary material for: Endurance swimming performance and physiology of juvenile Green Sturgeon (Acipenser medirostris) at different temperatures
Source: Conserv Physiol. 2025 Feb 8;13(1):coaf003. doi: 10.1093/conphys/coaf003 (PMC11815015; doi:10.1093/conphys/coaf003)
Supplement: Web_Material_coaf003 [file web_material_coaf003.zip › GS_endurance _phys supp mat_2024_revised.pdf]

Supplementary material

Sup. Table 1. Statistical tables of the glm models used to examine the effects of tank within each temperature treatment on the response variables: temperature (°C) and dissolved oxygen (mg l^{-1}).

Variable	Model	Temperature	df	f-value	*p*-value
Temperature (°C)	lm(temp_c~tank_id, data=data)	13°C	3	0.3222	0.8093
	lm(temp_c~tank_id, data=data)	18°C	3	0.0169	0.997
Dissolved oxygen (mg l^{-1})	lm(do_mgl~tank_id, data=data)	13°C	3	0.5058	0.6804
	lm(do_mgl~tank_id, data=data)	18°C	3	0.9979	0.4009

Sup Table 2. Daily water temperatures in the Sacramento river at the Red Bluff Diversion Dam by month from the California Data Exchange Center created by the California Department of Water Resources (cdec.water.ca.gov). Hourly temperature data from 2022 (the year of this study) also shown. Data are represented as means ± SD, [in brackets: ranges].

Month	Average temperature (°C) 2018-2022	Average temperature (°C) 2022	Temperature range (°C) [min – max] 2022
January	9.5 ± 0.9	8.8 ± 0.7	[7.1 ± 10.2]
February	9.6 ± 1.1	9.8 ± 1.1	[7.4 ± 12.3]
March	11.2 ± 1.3	12.5 ± 1.6	[9.8 ± 15.9]
April	13.6 ± 1.4	13.9 ± 1.5	[11.2 ± 17.6]
May	15.2 ± 1.5	16.6 ± 1.6	[12.6 ± 20.6]
June	15.6 ± 1.3	17.8 ± 1.3	[14.8 ± 20.4]
July	15.5 ± 1.3	17.8 ± 1.0	[15.8 ± 20.2]
August	15.1 ± 1.1	17.6 ± 0.9	[15.4 ± 19.7]
September	14.6 ± 1.8	16.5 ± 0.9	[14.6 ± 18.7]
October	13.6 ± 1.6	15.2 ± 1.2	[12.6 ± 17.4]
November	11.4 ± 1.9	11.7 ± 0.9	[9.6 ± 14.0]
December	9.7 ± 1.4	9.3 ± 0.8	[7.6 ± 10.8]

Sup. Table 3. Statistical tables of the multiplicative glm models used to examine the effects of either fixed water velocity (cm s^{-1}) or swimming type and temperature on the response variables: total length, fork length, and weight.

Variable	Model	Contrast	df	f-value	*p*-value
Total Length (cm)	lm(TL~Temperature*Velocity, data=data)	Temperature	1	0.074	0.786
		Velocity	7	0.346	0.931
		Temperature x Velocity	3	0.385	0.764
	lm(TL~Temperature*Swim.type, data=data)	Temperature	1	0.076	0.783
		Swimming type	3	0.817	0.487
		Temperature x Swimming type	3	0.032	0.992
Fork Length (cm)	lm(FL~Temperature*Velocity, data=data)	Temperature	1	0.481	0.489
		Velocity	7	0.300	0.953
		Temperature x Velocity	3	0.334	0.801
	lm(FL~Temperature*Swim.type, data=data)	Temperature	1	0.493	0.484
		Swimming type	3	0.643	0.588
		Temperature x Swimming type	3	0.033	0.992
Weight (g)	lm(Weight~Temperature*Velocity, data=data)	Temperature	1	0.002	0.963
		Velocity	7	0.176	0.990
		Temperature x Velocity	3	0.142	0.935
	lm(Weight~Temperature*Swim.type, data=data)	Temperature	1	0.002	0.962
		Swimming type	3	0.337	0.798
		Temperature x Swimming type	3	0.128	0.943

Sup Table 4. Statistical tables of the multiplicative lmer models used to examine the effects of either fixed water velocity (cm s^{-1}) or swimming type and temperature on the response variables: time-to-fatigue, whole-body glucose concentration, whole-body lactate concentration, whole-body cortisol concentration, and whole-body total protein.

Variable	Model	Contrast	df	f-value	*p*-value
Time-to-fatigue (min)	lmer(Time.to.end~ Velocity * Temperature + (1|Tunnel)	**Velocity**	**6**	**31.8**	**<0.0001**
		**Temperature**	**1**	**41.7**	**<0.0001**
		**Velocity x Temperature**	**2**	**10.0**	**0.0001**
Glucose (µg g^{-1})	lmer(Glucose~ Velocity * Temperature + (1|FL)	Velocity	7	1.2	0.2891
		Temperature	1	2.6	0.1072
		Velocity x Temperature	3	1.4	0.2401
	lmer(Glucose~ Swim type * Temperature + (1|FL)	Swimming type	3	2.2	0.0899
		Temperature	1	3.4	0.0671
		Swim x Temperature	3	0.5	0.6544
Lactate (µg g^{-1})	lm(log(Lactate)~ Velocity * Temperature	**Velocity**	**7**	**3.9**	**0.0008**
		Temperature	1	2.0	0.1590
		Velocity x Temperature	3	1.2	0.3025
	lm(log(Lactate)~ Swim type * Temperature	**Swimming type**	**3**	**8.5**	**<0.0001**
		Temperature	1	0.9	0.3374
		Swim x Temperature	3	2.6	0.0568
Cortisol (ng g^{-1})	lm(sqrt(Cortisol)~ Velocity * Temperature +(1|Tunnel)	Velocity	7	2.1	0.1135
		Temperature	1	2.6	0.1132
		**Velocity x Temperature**	**3**	**5.3**	**0.0019**
	lm(sqrt(Cortisol)~ Swim type * Temperature + (1|Tunnel)	**Swimming type**	**3**	**11.6**	**0.0018**
		Temperature	1	2.3	0.1294
		**Swim x Temperature**	**3**	**3.0**	**0.0344**
Total protein (µg g^{-1})	lmer(log(Protein)~ Velocity * Temperature	**Velocity**	**7**	**2.8**	**0.0097**
		**Temperature**	**1**	**30.3**	**<0.0001**
		Velocity x Temperature	3	0.5	0.6654
	lmer(log(Protein)~ Swim type * Temperature	Swimming type	3	0.3	0.8095
		**Temperature**	**1**	**36.6**	**<0.0001**
		Swim x Temperature	3	0.4	0.7703

Sup. Table 5. Statistical tables of the additive glmer models used to examine the probability of station holding compared to swimming type/BL s^{-1} and temperature and the probability of entrainment compared by BL s^{-1} and temperature.

Variable	Model	Contrast	chi-square statistic	df	*p*-value
Probability of station holding	glmer(Station.holding~Swim.type + Temperature + (1|Tunnel), family=binomial(link='logit')	**Swimming type**	**6.5**	**2**	**0.0394**
		Temperature	2.4	1	0.1243
	glmer(Station holding~Bl _s+Temperature + (1|Tunnel), family=binomial(link='logit')	**BL s^{-1}**	**13.3**	**1**	**0.0003**
		Temperature	0.9	1	0.3548
Probability of avoiding impingement	glmer(Prob.impingement~Bl _s+Temperature + (1|Tunnel), family=binomial(link='logit')	**BL s^{-1}**	**8.0**	**1**	**0.0048**
		**Temperature**	**21.1**	**1**	**<0.0001**

Sup. Table 6. Statistical tables of the post-hoc tests used to examine whole-body cortisol (ng g^{-1}), lactate (µg g^{-1}) and total protein (µg g^{-1}) analyzed by swimming type or velocity compared to control nested within temperature.

Response Variable	Model	Temperature	Contrast	df	t-ratio	*p*-value
Lactate	lm(log(Lactate)~ Swim type * Temperature	13°C	Prolonged - Control	112	0.529	0.9351
			Rapid - Control	112	0.769	0.8279
			Sustained - Control	112	-1.554	0.3255
		18°C	**Prolonged - Control**	**112**	**4.121**	**0.0002**
			**Rapid - Control**	**112**	**3.792**	**0.0007**
			Sustained - Control	112	2.041	0.1251
Cortisol	lm(sqrt(Cortisol)~ Swim type * Temperature + (1|Tunnel)	13°C	**Prolonged - Control**	**109**	**2.816**	**0.0172**
			Rapid - Control	109	1.342	0.4538
			Sustained - Control	109	2.349	0.0606
		18°C	**Prolonged - Control**	**109**	**7.693**	**<.0001**
			**Rapid - Control**	**109**	**3.733**	**0.0009**
			**Sustained - Control**	**109**	**4.101**	**0.0002**
Lactate	lm(log(Lactate)~ Velocity * Temperature	13°C	25 - Control	108	-2.56	0.0578
			30 - Control	108	-0.82	0.9309
			35 - Control	108	-0.295	0.9993
			40 - Control	108	0.783	0.9426
			45 - Control	108	0.807	0.9353
		18°C	35 - Control	108	2.382	0.0913
			40 - Control	108	1.928	0.2521
			45 - Control	108	2.406	0.0859
			**50 - Control**	**108**	**3.424**	**0.0044**
			**55 - Control**	**108**	**3.36**	**0.0054**
Cortisol	lm(sqrt(Cortisol)~ Velocity * Temperature +(1|Tunnel)	13°C	25 - Control	10.5	0.517	0.9916
			30 - Control	10.5	1.607	0.5235
			35 - Control	10.5	2.68	0.1061
			40 - Control	11.1	1.171	0.7872
			45 - Control	10.6	0.937	0.9005
		18°C	35 - Control	10.6	2.344	0.1840
			**40 - Control**	**11.2**	**3.274**	**0.0359**
			**45 - Control**	**10.5**	**4.306**	**0.0069**
			50 - Control	11.2	2.671	0.1031

Total protein	lmer(log(Protein)~ Velocity * Temperature		55 - Control	10.5	2.527	0.1366
		13°C	25 - Control	105	-0.822	0.9303
			30 - Control	105	-0.993	0.8577
			35 - Control	105	0.409	0.9968
			40 - Control	105	1.03	0.8381
			45 - Control	105	-0.196	0.9999
		18°C	35 - Control	105	-0.769	0.9466
			40 - Control	105	-0.33	0.9989
			45 - Control	105	-0.24	0.9998
			50 - Control	105	0.054	1.0000
			55 - Control	105	-2.102	0.1758
